# Patient-reported experiences of pulmonary fibrosis in the UK: The role of sex and disease subtypes in symptomology, diagnosis and care

**DOI:** 10.1177/17534666261492562

**Published:** 2026-09-23

**Authors:** Georgie May Massen, Lisa Murray, Nazia Chaudhuri, Bradley Price

**Affiliations:** 1 School of Public Health, 4615 Imperial College London, London, England; 2 The George Institute, 4615 Imperial College London, London, England; 3 Action for Pulmonary Fibrosis, Peterborough, England; 4 School of Medicine, Department of Life and Health Sciences, Ulster University, Londonderry, Northern Ireland, UK; 5 Altnagelvin Area Hospital, Western Health and Social Care Trust, Londonderry, Northern Ireland, UK

**Keywords:** Patient care, pulmonary fibrosis, interstitial lung disease

## Abstract

**Background:**

Pulmonary fibrosis (PF) is associated with substantial symptom burden, complex diagnostic pathways and varied access to specialist services. It is important to understand patient and carer experiences to highlight disparities and inform improvements in care.

**Objectives:**

To explore reported insights from patients and carers regarding PF symptomology, initial healthcare experiences, access to support services, and whether experiences differ by sex and PF subtype.

**Design:**

A cross-sectional analysis of the 2025 PF State of the Nation Survey, conducted by Action for Pulmonary Fibrosis.

**Methods:**

Using the survey results which included information from 1,270 individuals (73.78% patients) with lived experience of PF. Differences in symptoms, diagnostic pathways and patient experience were examined using Chi-squared and Fishers exact tests.

**Results:**

Respondents with idiopathic pulmonary fibrosis (IPF) were more likely to be male, whilst female dominance was observed in those reporting diagnosis of non-IPF-PF. Males were diagnosed at an older age than females, were referred to respiratory services more quickly, and had fewer primary care appointments prior to referral. More than 90% of the cohort reported symptoms prior to diagnosis, but symptomology differed by PF subtype; breathlessness was more commonly reported by those with hypersensitivity pneumonitis (p=0.004) and cough was more commonly reported by people with sarcoidosis (p=0.025). Almost one quarter (24.93%) of people reported symptoms to a physician only after experiencing them for at least one year. Treatment burden was high, antifibrotic side effects were common and differences with respect to age and sex were observed.

**Conclusions:**

Important differences in symptom burden, diagnostic experiences and access to support exist across PF subtypes and between sexes. These findings highlight potential weaknesses in current diagnostic pathways, as well as unmet needs related to referral delays, treatment side effects and support services. Enhancing patient-centred care and targeted service provision may help to reduce inequalities and improve outcomes.

## Plain language summary

Why was this study done?

Pulmonary fibrosis (PF) is a group of serious lung diseases that cause permanent scarring of the lungs. Although treatments and support are available, many people face delays in diagnosis and unequal access to specialist care. We wanted to understand the experiences of people affected by PF and whether these differed between women and men or between different types of PF.

What did the researchers do?

We analysed responses from 1,270 people across the UK who were living with PF, caring for someone with PF, or had been bereaved by the disease. We looked at symptoms, diagnosis, treatment experiences and access to support services across PF subtypes and across males and females.

What did the researchers find?

More than 9 in 10 people had symptoms before they were diagnosed. Around 1 in 4 waited a year or more before telling a healthcare professional about their symptoms, and around 1 in 3 said they had been given another diagnosis before PF was identified. Women were more likely than men to experience delays in being referred to a lung specialist, needed more GP appointments before referral, and were less likely to feel their concerns had been taken seriously. Many people also struggled to access services such as pulmonary rehabilitation, psychological support and advance care planning, even when they believed these would have helped.

What do these findings mean?

Our findings suggest that many people with PF are not receiving timely, equitable care. Improving awareness of PF, reducing delays to specialist referral, recognising differences in how PF affects people, and ensuring better access to support services could improve care and quality of life. Because this study was based on people’s own reports rather than medical records, some details could not be clinically confirmed and may not always have been recalled or recorded fully accurately.

## INTRODUCTION

Pulmonary fibrosis (PF) describes a group of chronic progressive interstitial lung diseases which are characterised by the presence of inflammation and fibrosis within the lung parenchyma. Interstitial lung disease (ILD) subtypes are categorised by aetiology, presentation and prognosis. The most common PF is idiopathic pulmonary fibrosis (IPF). Pulmonary fibrosis may also arise in connection with connective tissue diseases (such as rheumatoid arthritis) or as a result of exposures (including environmental, drug and radiation exposure).^1,2^ Due to the varied aetiology of ILDs, the available treatment differs; some may benefit from immunosuppressive medications, whilst others may be eligible for antifibrotic treatment, depending on the driver of their disease.^3^

People with PF can experience a variety of symptoms, including breathlessness, cough, unexplained weight loss and fatigue, however symptoms vary by subtype as well as disease stage.^4^ Due to the progressive nature of PF, poor prognosis, inevitable lung function decline and eventual respiratory failure, patients should have access to a multitude of healthcare services to support them in managing and living with their condition. These services may include pulmonary rehabilitation, access to supplemental oxygen, dietary guidance, psychological support and end of life planning.^5^

Despite improvements in epidemiological evidence of PF, gaps in the knowledge base remain with respect to disease symptomology and how this differs between subtypes as well as sex. It is known that side effects of current antifibrotic medication can lead to early discontinuation and may negatively impact quality of life due to gastrointestinal problems and fatigue.^6–8^ It is not currently well understood if side effects of antifibrotics differ by age or sex. Systematic, representative and well-designed research is pivotal to guide understanding of symptomology and treatment plans leading to improved care for people with PF.

The 2025 Pulmonary Fibrosis State of the Nation Survey was designed by the UK charity Action for Pulmonary Fibrosis (APF), with the aim of gathering the perspectives of patients, carers and bereaved individuals with respect to perceived experiences of PF care. The methods of survey development are available in Supplemental Appendix 1a. A further aim was to highlight areas of strength and weakness along the care pathway, to better inform policy and guide change in the way those affected by PF interact with healthcare providers. The objective of this manuscript was to describe the symptom burden, pathway to diagnosis and treatment side effects and how these differed by sex. Additionally, we describe the provision and perceived need of supportive services.

## METHODS

### Dataset

The Pulmonary Fibrosis State of the Nation lived experience Survey was deployed in four languages (English, Welsh, Polish and Punjabi) on the 21/05/2025 and closed submissions on the 30/06/2025.^9^ This survey was made available both in paper and online formats and could be completed over the phone with APF staff. The survey was distributed via a range of channels, with additional actions taken to reach ‘cold audiences’ unfamiliar with APF. It was emailed to ILD networks and respiratory clinicians who were requested to share this with their patients and additionally it was shared via LinkedIn posts and emails with clinical, corporate and charity partners who provided services to people who are affected by PF. More broadly the survey was promoted within the APF supporter newsletter and magazine, details of the survey were shared on social media platforms and within support groups. Physical copies were available in English within ILD clinics and support groups and could be posted directly to anyone who requested it. Proportion of missing data can be viewed in Supplemental Appendix 1b. The survey was self-reported and briefly covered the following themes: symptoms, diagnosis, misdiagnosis and being heard, antifibrotic treatment, symptom management and support services and access to specialist care. The survey questions are available in Supplemental Appendix 1c.^10^

The collected data were systematically cleaned prior to analysis. Multiple submissions from the same respondent were identified, where responses were found to be identical the duplicates were removed, where responses differed, the later of the submissions (identified from time stamps) were retained. Additional free-text was examined to understand the wider context prior to deduplication. Responses from people outside of the UK were removed and responses from professionals or experts who did not identify that they had a direct relationship with someone with pulmonary fibrosis were removed. All free-text records were standardised for spelling to ensure accurate grouping of responses. Data were anonymised prior to analyses. To ensure accurate statistical analysis and avoid the potential double-counting of individuals with PF, responses from carers were excluded from analyses involving clinical or demographic variables (such as diagnosis and age). Only responses from patients and bereaved individuals were retained in these analyses. However, responses from carers were included when analysing self-reported experiences of care (for example, whether respondents felt listened to or sufficiently informed).

### Pulmonary fibrosis subtypes

Where possible we describe the responses across self-reported subtypes of PF. These are: idiopathic pulmonary fibrosis (IPF), rheumatoid arthritis associated interstitial lung disease (RA-ILD), connective tissue disease associated interstitial lung disease (CTD-ILD), hypersensitivity pneumonitis (HP), COVID-19 interstitial lung disease, idiopathic interstitial pneumonia (IIP), occupational PF (which included silicosis and asbestosis), pulmonary sarcoidosis and Other PF (including combined pulmonary fibrosis and emphysema (CPFE), drug induced PF (D-ILD), familial pulmonary fibrosis, fibrosing organising pneumonia, radiation induced pulmonary fibrosis and progressive pulmonary fibrosis). We also compared the perspectives of people with IPF with those of people with non-IPF-PF. Survey responders were also able to provide information regarding disease subtype using free text which was subsequently used to inform grouping. Whilst we use the term non-IPF-PF throughout this manuscript we cannot confirm that these conditions are progressive in nature and therefore avoid progressive pulmonary fibrosis terminology.

### Analyses

Continuous variables are reported as mean±SD or median with IQR as appropriate, while categorical variables are presented as numbers and percentages. Group comparisons are conducted using χ^2^ tests for categorical variables, independent t-tests for normally distributed continuous variables and Mann-Whitney U tests for skewed continuous data. A p-value<0.05 was considered statistically significant.

We described the distribution of respondents across pulmonary fibrosis (PF) subtypes and examined differences by sex (male/female) and age at diagnosis (25 or under, 26-25, 36-45, 46-55, 56-65, 66-75, 76+). The proportion of respondents reporting symptoms prior to PF diagnosis was summarised, including both the number of symptoms reported and the prevalence of individual symptoms, stratified by PF subtype and sex. Differences between groups were formally tested.

We assessed the average time between first experiencing symptoms and first reporting them to a primary care physician (less than 3 months, between 3 and 6 months, between 7 and 12 months, between one and two years, more than two years) and subsequently stratified results by sex and PF subtype. Routes of access to care were summarised as the first healthcare service to which symptoms were reported (GP, community diagnostic centre, lung cancer screening, a different speciality, respiratory services, private healthcare, accident and emergency/urgent care), with the proportion of respondents using each route described and sex differences explored.

Among respondents who initially reported symptoms to a primary care physician, we examined the average time to referral and the number of primary care visits related to symptoms prior to referral, comparing results across PF subtype and sex. We also reported the proportion of respondents who self-reported a prior misdiagnosis before receiving a PF diagnosis, stratified by PF subtype, and compared misdiagnosis rates with those observed among individuals with IPF and non-IPF-PF respectively. Respondents were asked if they had been misdiagnosed and if so, what those misdiagnoses were. These were manually cleaned to ensure only conditions which were not PF were retained. Sex differences in misdiagnosis rates within each PF subtype were explored. Additionally, we described the proportion of respondents who received treatment for the condition they were misdiagnosed with and assessed whether treatment receipt differed by sex.

Throughout this report some analyses were conducted using only the data of people with current PF (patients) to minimise bias, whilst no restrictions were applied for other questions. Where necessary the specific group of people whose data were analysed is clearly stated.

All analyses were descriptive and unadjusted and were intended to compare patient-reported experiences between groups rather than identify independent predictors of outcomes.

All analyses were undertaken using Stata version 17^10^. This manuscript was prepared in accordance to STROBE guidelines (Appendix 1d).^11^

## RESULTS

### Respondent demographics

In total, the opinions of 1,270 individuals were gathered. 937 (73.78%) were currently living with PF, three people had received a lung transplant for their PF (0.2%), whilst 154 people (12.13%) were carers of people with PF and 176 (13.86%) were bereaved carers of people with PF. The majority of responses were from people living in England (78.58%), with 9.68% of responses from individuals in Scotland and 7.53% from Wales and 2.60% from Northern Ireland (Supplemental Table 1). Using the data of individuals with pulmonary fibrosis and bereaved individuals, the most common PF subtype was IPF (n=728, 65.23%) (Figure 1). 53.14% of respondents were male and male dominance was observed in IPF (59.89%, p<0.001) and occupational PF (76.92%, p=0.0027). Non-IPF-PF was more common in females (58.51%, p<0.001), as was hypersensitivity pneumonitis (67.57%, p=0.0025), CTD-ILD (67.19%, p=0.0038), RA-ILD (63.64%, p=0.043), and PF of unknown subtype (64.44%, p=0.009). Age distribution at diagnosis varied by PF subtype and sex. Age distribution at diagnosis differed significantly between IPF and non-IPF-PF subgroups (p<0.001), with IPF more commonly diagnosed at an older age (p<0.001) (Supplemental Table 2). Males with IPF were diagnosed at an older age compared with females (p=0.0023), this was also observed in the cohort of people with non-IPF-PF (p=0.0265) and hypersensitivity pneumonitis (p=0.0294), but not other PF sub-types (Supplemental Table 3).

**Figure 1.**
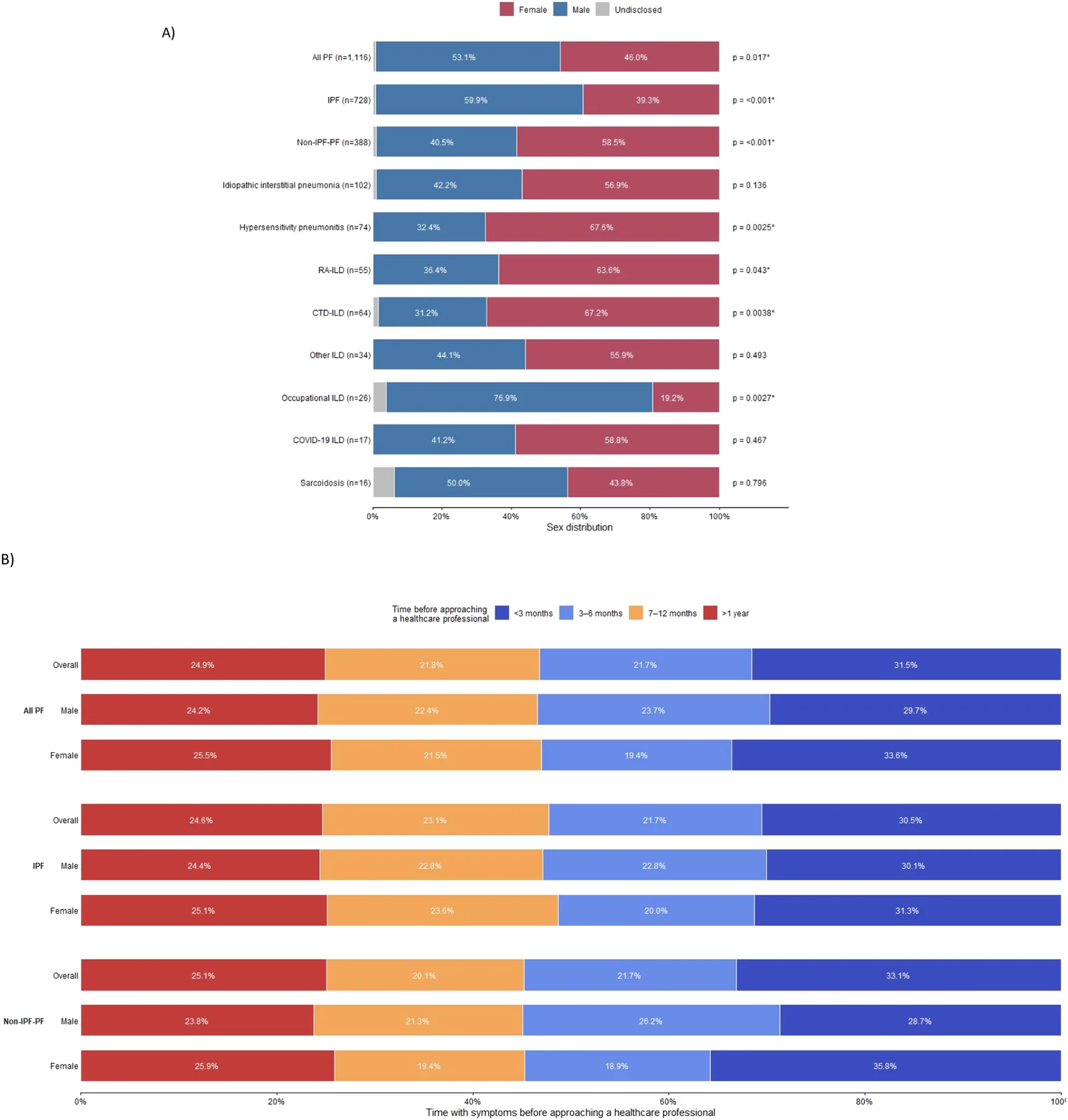
A) Sex distribution by pulmonary fibrosis subtype. B) Time before approaching healthcare professional, stratified by sex and pulmonary fibrosis subtype. Using the data of individuals with PF.

### Symptom burden by ILD sub-type and sex

Of the people currently living with PF, 90.32% reported symptoms prior to diagnosis, sex difference was not observed with regards to number of symptoms for all PF sub-types (Supplemental Table 4). Differences in reported symptoms were observed across self-reported PF subtypes. Compared with IPF, breathlessness was significantly more common in people with HP (82.61% versus 65.63%, p=0.004) and cough was significantly more reported by people with sarcoidosis (84.62% vs 52.85%, p=0.025) (Supplementary Figure 1). Fatigue was more commonly reported by those with COVID-19 related PF (56.25% versus 29.19%, p=0.027) and sarcoidosis (61.54% versus 29.19%, p=0.026) compared with IPF. Unexplained weight loss was more common in those with RA-ILD (16.33%, p=0.0012) and sarcoidosis (23.08%, p=0.014) compared with people with IPF (3.80%).

Symptoms also differed by sex; in people with non-IPF-PF, a greater percentage of males reported breathlessness compared with females (79.86% vs 69.72%; p=0.037), which wasn’t the case in IPF or other PF sub-types (Supplemental Table 5). Similarly, a significantly greater percentage of males with RA-ILD reported experiencing breathlessness compared with females (93.33% vs 55.88%, p=0.002) and unexplained weight loss was more commonly observed in males with IIP compared with females (13.16% vs 1.75%, p=0.036).

31.53% of people reported their symptoms to a healthcare professional within the first three months following onset however, 24.93% of people reported symptoms after experiencing them for at least twelve months. No statistical difference in the time between developing symptoms and reporting symptoms was observed when comparing sexes in all PF subtypes, IPF cases or non-IPF-PF cases (Figure 2). Examining the data of people living with PF and bereaved individuals revealed that 73.01% of people first reported symptoms to a general practitioner whilst 7.12% presented at either Accident and Emergency departments or urgent care centres, route of access did not differ by sex (Table 1).

**Figure 2.**
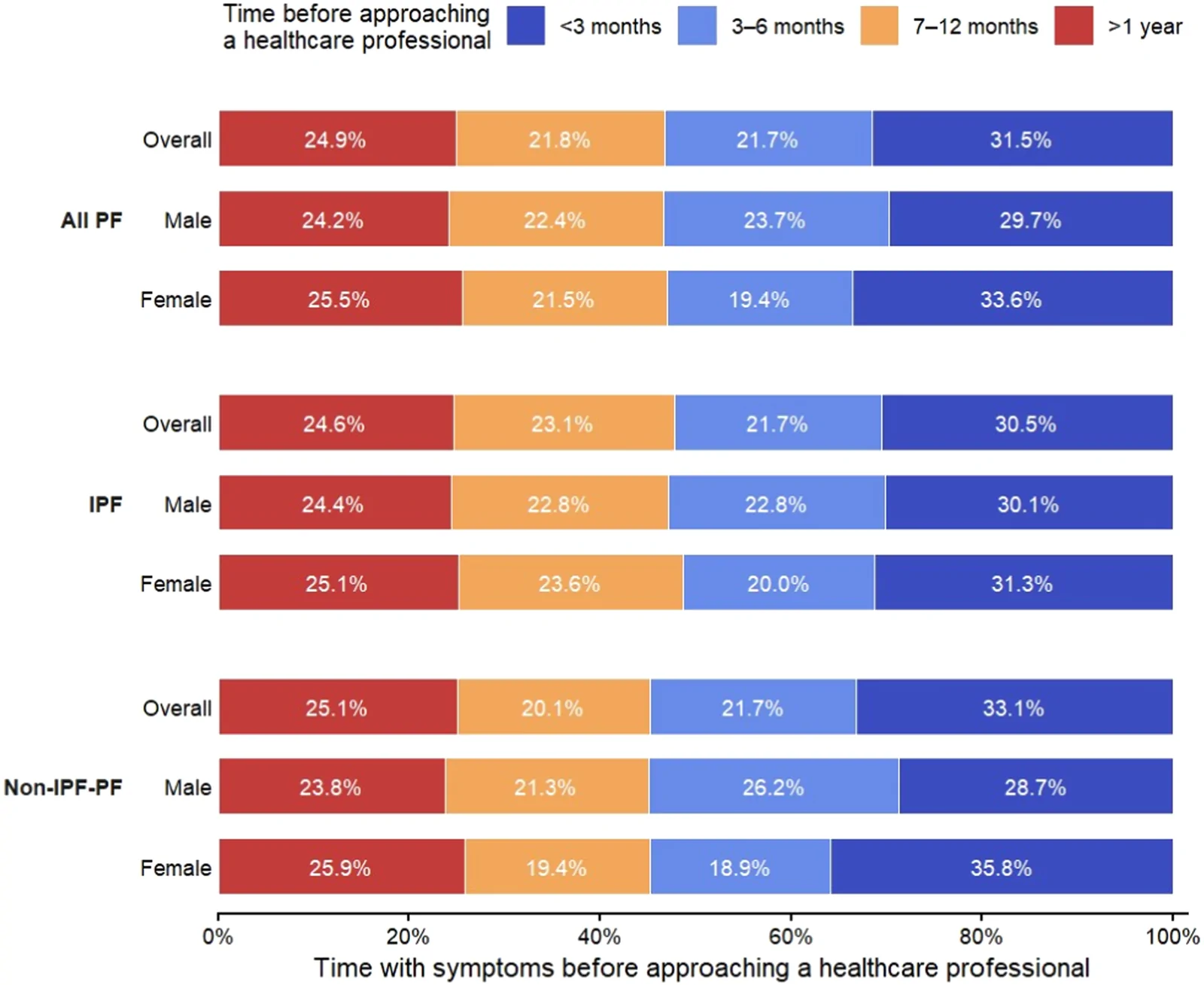
Average time between developing symptoms and reporting symptoms by sex and broad pulmonary fibrosis subtypes. Using the data of individuals with PF.

**Table 1.** Initial route of access by sex. Using the data of people with pulmonary fibrosis.

Route of access	Percentage (%)			p-value
	All	Male	Female	
A&E/Urgent care	7.48	7.95	7.05	0.051
General practice	75.37	73.25	77.31	
Referral from another specialist	8.29	10.36	6.39	
Respiratory service	4.49	4.10	4.85	
Private healthcare	4.37	4.34	4.41	

This analysis did not include the responses from 34 individuals as they were unable to report the initial point of contact or did not wish to report sex.

### Health care referral

Of the people who reported symptoms to their primary care physician, 33.98% were referred to be seen by a respiratory specialist within one month whilst 14.42% waited more than one year before being referred (Table 2). It was observed that people with CTD-ILD approached their primary care physician more frequently prior to referral regarding their symptoms compared with people with IPF (p=0.029). There was a significant difference with respect to sex and time between reporting symptoms and referral, using the data of all individuals. Males were referred significantly quicker than females (p<0.001). This finding was also observed in those with IPF, females reported a significantly longer time to referral than males (p<0.001). Additionally, females presented to their primary care physician with symptoms significantly more times than males before being referred to a respiratory specialist (all-PF, p<0.001, non-IPF-PF, p<0.001) (Supplemental Table 6). Males with HP reported significantly less visits to their primary care physician before being referred compared with females (p=0.006). 18.78% of people currently living with PF did not feel that their health concerns were listened to or taken seriously; statistically significant sex differences were observed in responses with a higher proportion of females reporting that concerns were not listened to or taken seriously to compared with males (p<0.001) (Supplemental Table 7). 36.07% of people currently living with PF believed they experienced avoidable delays (Supplemental Table 8); no statistically significant difference in responses was observed between sexes (p=0.17).

**Table 2.** Time between contacting general practitioner and being referred to a respiratory specialist in those whose route of access was primary care.

​	Sex	< 1 month	1-3 months	3-6 months	6- 12 months	>1 year	p-value
All PF	​	33.98	26.91	15.40	9.29	14.42	​
	M	36.71	28.16	15.19	6.96	12.97	<0.001*
	F	30.58	25.21	16.12	14.05	14.05	
IPF	​	36.25	27.19	14.66	8.35	13.65	​
	M	39.75	27.20	14.23	5.86	12.97	<0.001*
	F	29.71	28.26	13.77	15.22	13.04	
Non-IPF-PF	​	29.13	26.52	16.96	11.30	16.09	​
	M	27.27	31.17	18.18	10.39	12.99	0.051
	F	31.73	21.15	19.23	12.50	15.38	
Covid-19 ILD	​	33.33	33.33	0.00	0.00	33.33	​
	M	100.00	0.00	0.00	0.00	0.00	0.059
	F	0.00	50.00	0.00	0.00	50.00	
CTD ILD	​	29.73	24.32	16.22	13.51	16.22	​
	M	46.15	0.00	30.77	7.69	15.38	0.067
	F	21.74	39.13	8.70	17.39	13.04	
Hypersensitivity pneumonitis	​	29.41	19.61	17.65	15.69	17.65	​
	M	23.53	41.18	5.88	17.65	11.76	0.053
	F	32.35	8.82	23.53	14.71	20.59	
Idiopathic interstitial pneumonia	​	28.79	27.27	18.18	10.61	15.15	​
	M	20.00	43.33	10.00	10.00	16.67	0.051
	F	34.29	14.29	25.71	11.43	14.29	
Occupational ILD	​	23.08	46.15	15.38	7.69	7.69	​
	M	20.00	50.00	20.00	0.00	10.00	0.11
	F	33.33	33.33	0.00	33.33	0.00	
Other ILD	​	15.00	30.00	20.00	0.00	35.00	​
	M	0.00	33.33	16.67	0.00	50.00	0.58
	F	21.43	28.57	21.43	0.00	28.57	
RA-ILD	​	43.75	28.12	15.62	6.25	6.25	​
	M	46.15	23.08	30.77	0.00	0.00	0.072
	F	42.11	31.58	5.26	10.53	10.53	
Sarcoidosis	​	12.50	25.00	12.50	37.50	12.50	​
	M	16.67	33.33	16.67	33.33	0.00	0.091
	F	0.00	0.00	0.00	50.00	50.00	

PF: pulmonary fibrosis, IPF: idiopathic pulmonary fibrosis, Non-IPF-PF: Non-idiopathic pulmonary fibrosis, ILD: Interstitial lung disease, CTD ILD: connective tissue disease associated interstitial lung disease, RA-ILD: rheumatoid arthritis associated interstitial lung disease.

M: males, F: females. * indicates p-value<0.05.

This analysis did not include responses from 17 individuals as they were unable to respond (do not know or prefer not to answer) to this question or did not report their sex.

### Misdiagnosis of PF

Misdiagnosis of PF was common (32.96%), affecting around one third of individuals with IPF (30.90%) and over one third of those with non-IPF-PF (35.75%) respectively (Supplemental Table 9). A significantly greater percentage of people with HP were misdiagnosed with another condition compared to those with IPF (43.06% vs 30.9%, p=0.046), this was also observed when comparing misdiagnoses in people with occupational PF (56.00%, p=0.014). No differences were observed between IPF and the remaining PF subtypes. No sex difference was observed with respect to the percentage of people who were misdiagnosed with another condition. Of those who were misdiagnosed, 70.82% received treatment for the misdiagnosed condition, sex differences were not observed with regards to treatment of misdiagnosed conditions. PF was misdiagnosed as a variety of conditions, including other chronic respiratory diseases, respiratory infections and cardiovascular disease. A large percentage of people who were misdiagnosed with cardiovascular problems were females (62.86%), though this was not statistically significant (Supplementary Table 10).

### Treatment burden and side effects

Of the people with PF who had received antifibrotic medications, 90.65% reported side effects (Supplemental Table 11). A wide range of side effects were reported, the most common was diarrhoea (reported by 58.40% of people), followed by decreased appetite (52.05%) and nausea (50.00%). Some side effects were more commonly reported in females, including nausea (63.73 vs 42.06%, p<0.001) vomiting (27.98% vs 11.76%, p<0.001), and headaches (20.73% vs 10.26%, p=0.0036). Diarrhoea was more prevalent in those who were younger at point of diagnosis (p=0.041) (Supplemental Table 12).

### Support service availability

Overall, 83.01% of respondents had access to information about PF, 53.32% received this information from a professional whilst 29.69% sourced this information from elsewhere (Figure 3). Healthcare professional referrals were most common for clinical and disease-management services, such as pulmonary rehabilitation (62.30%) and oxygen therapy (50.95%), while psychological support (18.59%) and advance care planning (19.42%) were least frequently referred to by health professionals. 41.72% of respondents had access to physiotherapy or occupational therapy whilst 34.51% could not access this service but believed it would have been beneficial. 25.76% of people had access to psychological support, however an additional 41.04% did not have access to this service but believed it would have been beneficial. Oxygen services were accessed by 54.06% of respondents, although 38.38% reported being unable to access these services despite the potential benefits. 25.94% of people had access to end of life or advanced care planning whilst 52.75% of respondents could not access such services but believed they would have been useful. Using the information provided by bereaved individuals, 40.91% of respondents reported they were unable to access end of life or advanced care planning services but believed it would have been beneficial. 67.81% of respondents stated their loved one was unable to access psychological support, but it would have been beneficial.

**Figure 3.**
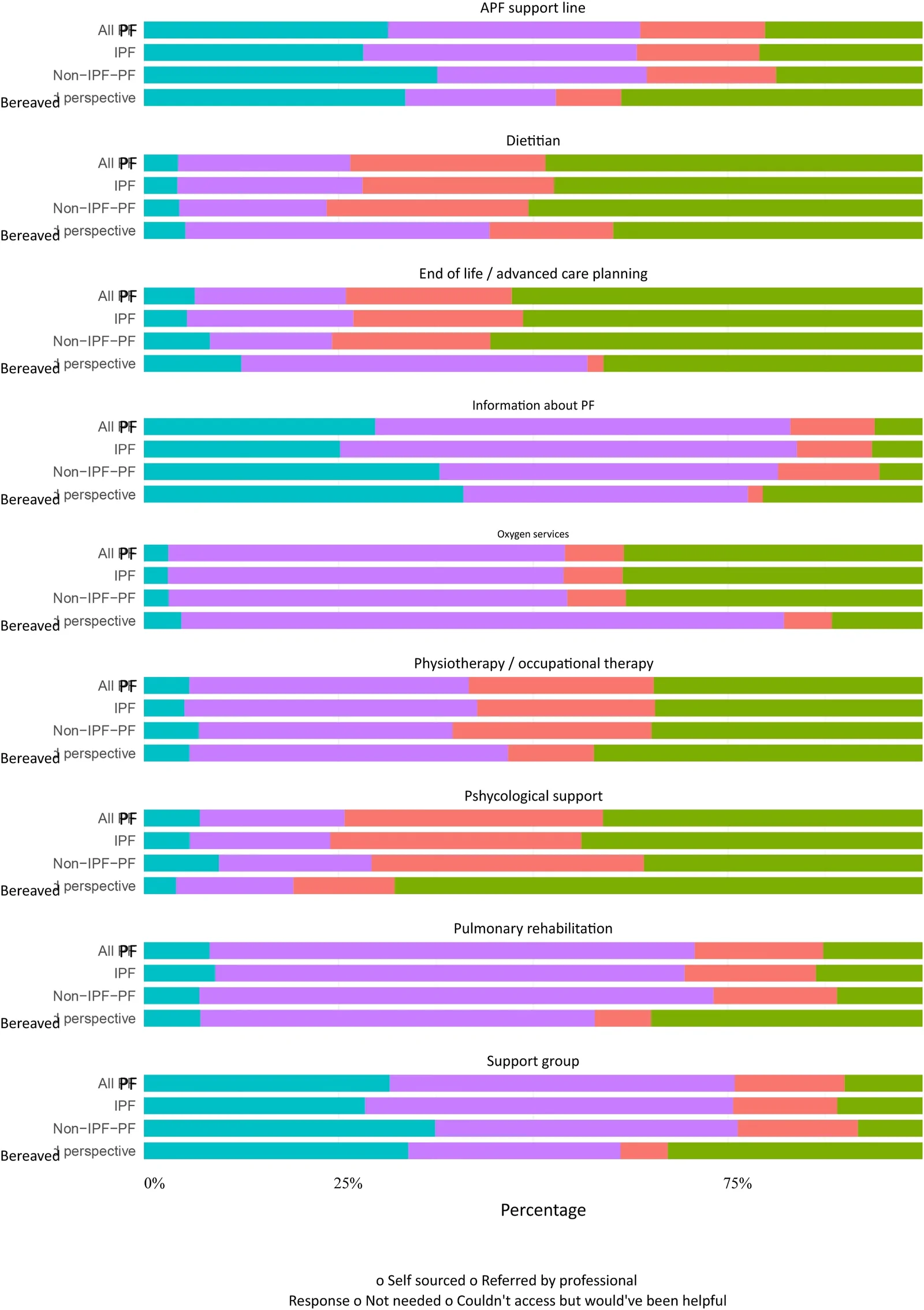
Access to support services, reported per broad pulmonary fibrosis subtype and by bereaved individuals. PF: pulmonary fibrosis, IPF: idiopathic pulmonary fibrosis, non-IPF-PF: non-idiopathic pulmonary fibrosis.

## Discussion

We have summarised the experiences of people living with pulmonary fibrosis and their carers, focusing on symptomology, experience of early diagnostic pathways and access to support services across PF subtypes. Patient-reported symptom burden differed according to self-reported pulmonary fibrosis subtype and by sex. Females reported needing to consult their primary care physician more frequently prior to referral. A large majority of people with IPF who had been prescribed antifibrotics experienced side effects; sex and age differences with respect to side effects were observed. Although many respondents believed support services would be beneficial, a large proportion were unable to access them.

Sex differences have historically been overlooked in medical research, however, increasing emphasis is now being placed on these important biological and structural differences, with policies now adopted by major funding bodies.^12^ Marked sex differences were observed across PF subtypes, including age at diagnosis and experience within diagnostic pathways. Male predominance in IPF and occupational PF aligns with prior studies, the female dominance in non-IPF-PF was also noted, particularly in CTD-ILD and RA-ILD. This may reflect sex specific risk of autoimmune diseases and exposure risk.^13–15^ Males were diagnosed at an older age compared with females; this may be indicative of sex specific differences in age at disease onset, disease trajectory, health-seeking behaviour or healthcare engagement. Sex differences in symptomology were also noted; these may be reflective of differences in breathing mechanics and lung structure.^16,17^ No sex difference was observed in time between developing and reporting symptoms, however females reported significantly longer delays between first presenting to healthcare and being referred for further investigation. In addition, females also needed to attend primary care more times than males and females were less likely to feel that their concerns were listened to.

Although misdiagnosis rates did not differ by sex, misdiagnosis patterns varied with females being more likely to be misdiagnosed with cardiovascular conditions. Misdiagnosis was more common among respondents with HP than IPF. This is biologically and clinically plausible, HP diagnosis requires integration of detailed environmental and occupational exposure history with radiological and multidisciplinary assessment. Overlap in radiological features with other fibrotic ILDs, further increases diagnostic complexity. Recent multicentre evidence suggests that careful evaluation of high-resolution CT features, particularly air trapping and centrilobular nodules, improves diagnostic accuracy and may facilitate earlier diagnosis.^18^ These findings highlight the importance of comprehensive exposure assessment and specialist radiological expertise within multidisciplinary ILD services. As pulmonary fibrosis subtype was self-reported rather than clinically verified, these subtype-specific findings should be interpreted cautiously, although they are consistent with the recognised diagnostic complexity of HP. These findings suggest there may not only be biological differences but also inequities in symptom recognition, recording of symptoms and escalation within diagnostic pathways.

When exploring the tolerability of antifibrotic medications, it was noted that females reported significantly greater side effect burden (including gastrointestinal symptoms and headaches) compared with males. This highlights important sex differences in drug tolerability, indicating that BMI and body composition should be considered when calculating appropriate drug dosage. Given that nausea and vomiting related to antifibrotic therapy can often be effectively managed with medications and supportive strategies, these findings raise concern that female patients may be disproportionately affected by potentially modifiable adverse effects.

Access to support services was frequently limited, with many respondents reporting unmet needs because of poor information sharing regarding support services as well as lack of referrals to support services. This may suggest that support is often introduced too late or inconsistently, rather than embedded throughout the disease course or from the point of diagnosis.

Existing studies have highlighted the short-term benefits of pulmonary rehabilitation (PR) in those with IPF. An exercise-based program like PR has been shown to improve exercise endurance, lung function, and dyspnoea in RCTs.^19,20^ Despite lack of evidence of longer-term impacts, multiple studies and meta-analyses have acknowledged the impact of physical activity on overall health, symptom management and quality of life during programme timeframes.^21,22^

Those that receive ILD-specific PR as part of multi-disciplinary care with community allied health support (nurse practitioner, respiratory therapist, occupational therapist, physiotherapist, and registered dietitian), are 24.2 times less likely to need respiratory-related urgent care.^23^ Limited access to services such as psychological support, rehabilitation, oxygen provision, and advance care planning may exacerbate symptom burden and contribute to inequities in care.^24–27^

Palliative care information and referral was particularly low, despite being a key provider of these supportive services, previously it has been shown that only 19.40% of people with IPF in England were referred to palliative care.^28^ This may reflect persistent perceptions among both clinicians and patients that palliative care is synonymous with end-of-life care, rather than a supportive, proactive approach that can be introduced alongside disease-directed treatment. Reframing palliative care as a positive step focused on symptom management, psychosocial support, and quality of life may help facilitate earlier engagement and reduce unmet need.^29,30^ Embedding structured referral to supportive services within standardised pulmonary fibrosis pathways may help ensure more timely and holistic support.

These findings highlight opportunities to improve PF care through changes in clinical practice, particularly within primary care. Enhanced education for healthcare professionals on early signs and symptoms of pulmonary fibrosis (highlighting sex differences) may lead to earlier disease recognition and therefore less primary care burden due to quicker referrals to specialist services. This may lead to diagnoses being made at a less severe disease stage, therefore improving treatment options. It is important that clinicians understand that pulmonary fibrosis does not only affect older males and instead can affect both males and females as well as younger individuals. Greater recognition of the diverse presentations of PF may lead to more equitable access to specialist services within shorter timeframes.

To address these issues and make the necessary changes in clinical practice, policy frameworks must be put in place that explicitly recognise and facilitate advancement in these priority areas. Investment in education and training for primary care physicians and other healthcare professionals is essential to ensure early diagnosis of pulmonary fibrosis. To enable effective implementation of standardised care pathways, instruments such as updated service specifications and clinical audits should be prioritised across Integrated Care Boards, Health Boards and NHS Regions. It is also essential to ensure that efforts are made to minimise regional variation in diagnostic timelines, treatment availability and support services. Effective care pathways will ensure equitable access to specialist care across the UK. The OneVoice ILD integrated clinical care pathway makes recommendations for a new model of care that would address the systemic access challenges and inequalities in PF care in England.^31^ Policy and research funding should prioritise further investigation into sex differences in pulmonary fibrosis, including disease progression and symptomology, to support more personalised and equitable care.

### Strengths and limitations

The study achieved the largest response to date for a UK-based survey of lived experience with PF, providing a broad UK sample and supporting the generalisability of the findings within the UK PF population. Inclusion of both patients and carers also provided a broader perspective on the diagnostic journey.

However, several limitations should be considered. The survey relied on self-reported data without independent clinical verification of diagnosis or PF subtype, introducing potential for misclassification and recall bias. Age at diagnosis was reported using categories rather than continuous age, which limits analytical precision. As this was a descriptive survey, multivariable adjustment was not undertaken; therefore, observed sex differences should not be interpreted as independent effects of sex and may reflect differences in PF subtype, age, disease severity, healthcare utilisation or other unmeasured factors. The survey was distributed primarily through Action for Pulmonary Fibrosis networks, respiratory services and support groups, which may have introduced selection and volunteer bias, while inclusion of only individuals alive and able to participate may have resulted in survivorship bias. Consequently, the findings may not be fully representative of the wider UK PF population. Furthermore, while overall response numbers were large, several pulmonary fibrosis subtypes had relatively small sample sizes, especially when analyses were stratified by sex, resulting in limited statistical power and restricting the use of formal hypothesis testing in some subgroups. As a result, some findings should be interpreted as descriptive rather than definitive.

## CONCLUSION

This study highlights important sex differences in self-reported pulmonary fibrosis, including differences in symptomology, diagnostic pathways, healthcare interactions and treatment tolerability. Compared with males, females reported longer delays to specialist referral and a greater burden of treatment-related side effects. More broadly, people living with pulmonary fibrosis continue to face challenges in accessing appropriate support services, driven by limited awareness of service availability and misconceptions among clinicians regarding when and for whom these services should be introduced.

## Supplemental Material

Supplemental Material - Patient-reported experiences of pulmonary fibrosis in the UK: The role of sex and disease subtypes in symptomology, diagnosis and care Supplemental Material for Patient-reported experiences of pulmonary fibrosis in the UK: The role of sex and disease subtypes in symptomology, diagnosis and care by Georgie May Massen, Lisa Murray, Nazia Chaudhuri, Bradley Price in Therapeutic Advances in Respiratory Disease

## Data Availability

Data used in this analysis are stored on a secure server and are not publicly available. Should you wish to use this data please contact Action for Pulmonary Fibrosis.

## References

[1] Travis WD Costabel U Hansell DM , et al. An official American Thoracic Society/European Respiratory Society statement: Update of the international multidisciplinary classification of the idiopathic interstitial pneumonias. Am J Respir Crit Care Med 2013; 188: 733–748. 10.1164/rccm.201308-1483ST PMC5803655 24032382

[2] Ryerson CJ Adegunsoye A Piciucchi S , et al. Update of the international multidisciplinary classification of the interstitial pneumonias: an ERS/ATS statement. Eur Respir J 2025; 66: 2500158. 10.1183/13993003.00158-2025 40774805

[3] Johnson SR Bernstein EJ Bolster MB , et al. 2023 American College of Rheumatology (ACR)/American College of Chest Physicians (CHEST) Guideline for the Treatment of Interstitial Lung Disease in People with Systemic Autoimmune Rheumatic Diseases. Arthritis Care Res (Hoboken) 2024; 76: 1051–1069. 10.1002/art.42861 PMC12646475 38973731

[4] Carvajalino S Reigada C Johnson MJ , et al. Symptom prevalence of patients with fibrotic interstitial lung disease: a systematic literature review. BMC Pulm Med 2018; 18: 78. 10.1186/s12890-018-0651-3 PMC5964639 29788938

[5] Kirby T . Marlies Wijsenbeek—advocating home monitoring in ILD. The Lancet Respiratory Medicine 2023; 11: 24–25. 10.1016/s2213-2600(22)00400-3 PMC9531919 36206782

[6] Oishi K Hirano T Murata Y , et al. Medication persistence rates and predictive factors for discontinuation of antifibrotic agents in patients with idiopathic pulmonary fibrosis: a real-world observational study, 2019. 10.1177/1753466619872890 PMC6719482 31476961

[7] Proesmans VLJ Drent M Elfferich MDP , et al. Self-reported Gastrointestinal Side Effects of Antifibrotic Drugs in Dutch Idiopathic Pulmonary Fibrosis patients. Lung 2019; 197: 551–558. 10.1007/s00408-019-00260-1 PMC6778529 31440832

[8] Lee J Park S Yoon H-Y . Effectiveness of antifibrotics on health-related quality of life in patients with interstitial lung disease: a systematic review and meta-analysis. Ther Adv Respir Dis 2025; 19: 17534666251390672. 10.1177/17534666251390672 PMC12580519 41174997

[9] Action for Pulmonary Fibrosis . PF State of the Nation Survey, actionpf, 2026. https://org/portals/0/page-content/documents/state-nation-survey-full-report.pdf

[10] StataCorp . Stata Statistical Software: Release 17. StataCorp LLC, 2021.

[11] von Elm E Altman DG Egger M , et al. The Strengthening the Reporting of Observational Studies in Epidemiology (STROBE) statement: guidelines for reporting observational studies. Lancet 2007; 370: 1453–1457. 10.1016/s0140-6736(07)61602-x 18064739

[12] MESSAGE-Policy-Framework.pdf. https://www.messageproject.co.uk/wp-content/uploads/2025/09/MESSAGE-Policy-Framework.pdf

[13] Mulholland S Dixon G Wells M , et al. Differences in disease characteristics and outcomes as determined by biological sex in a large UK idiopathic pulmonary fibrosis population: analysis from the British Thoracic Society, Interstitial Lung Disease registry data. BMJ Open Resp Res 2025; 12: e003301. 10.1136/bmjresp-2025-003301 PMC12481391 40998463

[14] Zaman T Moua T Vittinghoff E , et al. Differences in Clinical Characteristics and Outcomes Between Men and Women With Idiopathic Pulmonary Fibrosis. Chest 2020; 158: 245–251. 10.1016/j.chest.2020.02.009 PMC7339239 32084394

[15] Kawano-Dourado L Glassberg MK Assayag D , et al. Sex and gender in interstitial lung diseases. Eur Respir Rev 2021; 30: 210105. 10.1183/16000617.0105-2021 PMC9489177 34789464

[16] LoMauro A Aliverti A . Sex differences in respiratory function. Breathe (Sheff) 2018; 14: 131–140. 10.1183/20734735.000318 PMC5980468 29875832

[17] Townsend EA Miller VM Prakash YS . Sex Differences and Sex Steroids in Lung Health and Disease. Endocr Rev 2012; 33: 1–47. 10.1210/er.2010-0031 PMC3365843 22240244

[18] Shalmon T Freund O Wand O , et al. Hypersensitivity pneumonitis radiologic features in interstitial lung diseases. Respir Med 2025; 236: 107901. 10.1016/j.rmed.2024.107901 39631548

[19] Vainshelboim B Oliveira J Yehoshua L , et al. Exercise training-based pulmonary rehabilitation program is clinically beneficial for idiopathic pulmonary fibrosis. Respiration 2014; 88: 378–388. 10.1159/000367899 25341682

[20] Kataoka K Nishiyama O Ogura T , et al. Long-term effect of pulmonary rehabilitation in idiopathic pulmonary fibrosis: a randomised controlled trial. Thorax 2023; 78: 784–791. 10.1136/thorax-2022-219792 37012071

[21] Gaunaurd IA Gómez-Marín OW Ramos CF , et al. Physical Activity and Quality of Life Improvements of Patients With Idiopathic Pulmonary Fibrosis Completing a Pulmonary Rehabilitation Program. Respiratory Care 2014; 59: 1872–1879. 10.4187/respcare.03180 25185149

[22] Cheng L Tan B Yin Y , et al. Short- and long-term effects of pulmonary rehabilitation for idiopathic pulmonary fibrosis: a systematic review and meta-analysis. Clin Rehabil 2018; 32: 1299–1307. 10.1177/0269215518779122 29843523

[23] Kalluri M Claveria F Ainsley E , et al. Beyond Idiopathic Pulmonary Fibrosis Diagnosis: Multidisciplinary Care With an Early Integrated Palliative Approach Is Associated With a Decrease in Acute Care Utilization and Hospital Deaths. Journal of Pain and Symptom Management 2018; 55: 420–426. 10.1016/j.jpainsymman.2017.10.016 29101086

[24] Zou RH Kass DJ Gibson KF , et al. The Role of Palliative Care in Reducing Symptoms and Improving Quality of Life for Patients with Idiopathic Pulmonary Fibrosis: A Review. Pulm Ther 2020; 6: 35–46. 10.1007/s41030-019-00108-2 PMC7229085 32048243

[25] Micco A Carpentieri E Di Sorbo A , et al. Palliative care and end of life management in patients with idiopathic pulmonary fibrosis. Multidiscip Respir Med 2023; 18: 896. 10.4081/mrm.2023.896 PMC9994447 36909932

[26] Faverio P Bocchino M Caminati A , et al. Nutrition in Patients with Idiopathic Pulmonary Fibrosis: Critical Issues Analysis and Future Research Directions. Nutrients 2020; 12: 1131. 10.3390/nu12041131 PMC7231241 32316662

[27] Yamakawa H Ohta H Sato S , et al. Beneficial Impact of Nutritional Therapy on Idiopathic Pulmonary Fibrosis. Cureus 2025. 10.7759/cureus.78594 PMC11889364 40062119

[28] Morgan AD Khan H George PM , et al. Referral to pulmonary rehabilitation and palliative care services in people with idiopathic pulmonary fibrosis in England, 2010–2019. npj Prim Care Respir Med 2024; 34: 27. 10.1038/s41533-024-00387-6 PMC11464758 39384815

[29] Kreuter M Bendstrup E Russell AM , et al. Palliative care in interstitial lung disease: living well. The Lancet Respiratory Medicine 2017. 10.1016/S2213-2600(17)30383-1 29033267

[30] NICE . Quality statement 5: Palliative care | Idiopathic pulmonary fibrosis in adults. Quality standards. NICE. https://www.nice.org.uk/guidance/qs79/chapter/Quality-statement-5-Palliative-care2015

[31] Action for Pulmonary Fibrosis . Interstitial Lung Disease Care Pathway - One Voice ILD. https://actionpf.org/portals/0/page-content/documents/one-voice-ild-care-pathway-report.pdf (2024).

