# Supplementary material for: Patient-reported experiences of pulmonary fibrosis in the UK: The role of sex and disease subtypes in symptomology, diagnosis and care
Source: Ther Adv Respir Dis. 2026 Sep 23;20:17534666261492562. doi: 10.1177/17534666261492562 (PMC13735639; doi:10.1177/17534666261492562)
Supplement: Supplementary file 1 [file sj-pdf-1-tar-10.1177_17534666261492562.pdf]

Supplementary materials

Patient-Reported Experiences of Pulmonary Fibrosis in the UK: The role of sex and disease subtypes in symptomology, diagnosis and care.

G.M.Massen[1,2*], L.Murray[3*], N Chaudhuri[4], B.Price[3]

1. School of Public Health, Imperial College London, London, England

2. The George Institute, Imperial College London, London, England

3. Action for Pulmonary Fibrosis, Peterborough, England

4. School of Medicine, Department of Life and Health Sciences, Ulster University, Coleraine, Northern Ireland, UK

* Joint first authors

Supplementary Appendix 1a.

Survey Development Methodology:

- The survey was developed through a highly inclusive, patient-led process to ensure it reflected the real experiences and priorities of those affected by pulmonary fibrosis (PF).
- An initial focus group of 9 individuals—living with PF, caring for someone with PF, or bereaved—used a 'problem tree' method to explore the causes and impacts of key issues identified in the 2023 qualitative survey.
- These three core themes were then tested in additional focus groups, grouped by participants' relationship to PF: two with people living with IPF, one with PPF, and one with bereaved individuals (totalling 13 participants). All were invited to provide further feedback via email, one-to-one conversations, and to review and correct meeting minutes—ensuring everyone had a fair opportunity to contribute.
- Transcripts from all groups were analysed to identify shared concerns and divergent views, both within and across groups. This analysis informed the development of quantitative survey questions, focused on the most pressing and system-driven challenges.
- Draft questions were reviewed by a representative from each focus group for clarity, relevance, and accessibility. The survey then underwent internal review by the communications team and staff with lived experience of PF, before being piloted with 14 members of the Involvement Network.

Supplementary appendix 1b

High level description of variable missingness. A variable was defined as missing if a person did not disclose information as they were either unsure or preferred to not answer.

Variable	Valid responses (n)	Missing (n)	Missing (%)
Age at diagnosis	930	10	1.1
Sex	932	8	0.9
Time from symptom onset to reporting	861	79	8.4
First healthcare professional consulted	917	23	2.4
Time from GP consultation to respiratory referral*	642	17	2.6

*Question was only available for those whose route of access was via GP services

Supplementary appendix 1c

Pulmonary Fibrosis State of the Nation Survey

2025

Action for Pulmonary Fibrosis

Thank you for your interest in the Pulmonary Fibrosis State of the Nation survey. If you are a patient, carer, or bereaved individual, your views will help us understand what matters most to people affected by PF. These findings will help to show whether everyone is getting the care they need, and where improvements are needed. By sharing your experience, you can strengthen the campaign for better care from the NHS and improve the lives of others affected by PF.

You can also use the QR code or visit our website at www.actionpf.org to take the survey online.

Who is this survey for?

- People living with pulmonary fibrosis
- People caring for someone living with pulmonary fibrosis
- Bereaved caregivers of someone who had pulmonary fibrosis

If you are a carer or a bereaved carer, we will refer to the individual you care(d) as 'your loved one' throughout this survey. This is to keep questions to a readable length and does not mean to assume your relationship with them – we hope you understand.

How long will it take?

- The survey should take around 10-15 minutes
- We ask that you please complete the full survey, if possible. You can of course exit at any point, but we will only receive your answers if you click 'submit' at the end of the survey.
- A handful of questions cannot be skipped because they are branching questions that will take you to the next section most relevant to your experience.
- Any other questions you don't wish to answer can be left blank.

Please return the survey in the freepost envelope provided or send to:
Action for Pulmonary Fibrosis
Stuart House, East Wing, St John's Street,
Peterborough PE1 5DD

The online survey will close at midnight on 30th June 2025. If using freepost, please try to send on or before 30th June as responses received after 14th July unfortunately cannot be included.

Questions:
If you have any questions, please contact the policy team at policy@actionpf.org

Seeking Support:
Thinking back on your experience can be emotional. If you require any support after completing the survey, please consider reaching out to our support line at support@actionpf.org or by phoning 01223 785 725.

Printed Version:
If you require any additional printed versions of the survey, please email info@actionpf.org. If you would like to complete it over the phone, please call 01223 785 725.

Before you start

What will you use my answers for?
The survey will close on 30th June, following which we will analyse the responses. The findings will be shared publicly in a report later this year. The data we collect will help APF to build strong evidence that can be used to campaign for change in government policy and NHS care.

The data we publish will be anonymised, and your contact details will not be shared. All information you share with us will be stored securely.

By participating in this survey, you are agreeing to allow your anonymised answers to be published within a report as part of the survey results.

For more information on how we use and store data, please visit our privacy statement – www.actionpf.org/policies/privacy

2025 Pulmonary Fibrosis State of the Nation Survey

*Required

1 ☐ I understand that by completing this survey, I consent to my data being used as outlined above. (Please tick box)*

Your link to Pulmonary Fibrosis (PF)

The questions in this section are required to move onto the rest of the survey. This is because your answers to these questions will provide the context we need to understand your relationship to PF, and therefore your answers to the rest of the questions. 'Carer' also refers to giving help informally with care like transport to appointments etc.

2 What is your connection to pulmonary fibrosis:*

- ☐ I am living with pulmonary fibrosis
- ☐ I am a care giver for someone with pulmonary fibrosis
- ☐ I am a bereaved caregiver of someone with pulmonary fibrosis
- ☐ Other ______________________

3 What is the diagnosis of you or the person you care(d) for? If other, please specify:*

- ☐ Asbestosis
- ☐ Connective Tissue Disease ILD (CTD-ILD)
- ☐ Covid-19 related pulmonary fibrosis
- ☐ Hypersensitivity Pneumonitis (HP)
- ☐ Idiopathic pulmonary fibrosis (IPF)
- ☐ Idiopathic NSIP
- ☐ Rheumatoid Arthritis Interstitial Lung Disease (RA-ILD)
- ☐ Sarcoidosis
- ☐ Silicosis
- ☐ Systemic sclerosis ILD
- ☐ Occupational Interstitial Lung Disease (ILD)
- ☐ Don't know/can't remember
- ☐ Other ______________________

4 How long has it been since you or the person you care(d) for were diagnosed with PF?*

- ☐ Up to 6 months
- ☐ Between 6 months and 1 year
- ☐ More than 1 year and less than 2 years
- ☐ 2 – 4 years
- ☐ 4 - 6 years
- ☐ 6+ years
- ☐ Don't know/can't remember

First Symptoms

This section will ask about your/your loved one's experiences before starting the journey in PF care. This data helps us to understand and address the level of awareness of PF within the UK public.

5 Had you heard of PF before your diagnosis?

☐ Yes ☐ No ☐ Don't know/can't remember

Action for Pulmonary Fibrosis

6 What were the main health problems or symptoms that first suggested something might be wrong?

- [ ] Breathlessness
- [ ] Fatigue
- [ ] Other ____________
- [ ] Cough
- [ ] Unexplained weightloss

7 How long did you/your loved one have these symptom(s) for, before telling a healthcare professional?

- [ ] More than 1 month but less than 3 months
- [ ] 1-2 years
- [ ] More than 3 months but less than 6 months
- [ ] More than 2 years
- [ ] 7-12 months
- [ ] Don't know/can't remember

Diagnosis – Place of origin

This is a 'branching' question that will take you to the questions most relevant to your experience, based on your answer. Therefore, unfortunately this question cannot be skipped but it is to enable you to skip a section if it is not relevant to you. If you're not sure of the answer, please select 'don't know/can't remember' and continue to question 16.

8 Did your/your loved one's process towards a PF diagnosis start at the GP?*

- [ ] Yes – **go to Diagnosis – GP Entry, question 10**
- [ ] No – **go to Diagnosis – Other routes to diagnosis, question 9**
- [ ] Don't know/can't remember – **go to Diagnosis, misdiagnosis and being heard question 12**

Diagnosis – Other routes to diagnosis

Not everyone will start their PF journey at the GP. There are more opportunities to identify PF now than a few years ago and your response will help to build a better picture of how patients are currently entering the PF care pathway. This will help us to ensure that those on all routes of entry also receive appropriate support and quality of care.

9 If the journey toward a PF diagnosis did not begin with a GP visit, where did it begin instead? If not listed, please state in 'other'.

- [ ] Community Diagnostic Centre - testing/screening identified potential fibrosis
- [ ] Lung Cancer Screening - testing/screening identified potential fibrosis
- [ ] Referral from another specialist - e.g. rheumatology department, oncology department
- [ ] Respiratory Services - Initially misdiagnosed or had existing condition
- [ ] Used a private healthcare provider
- [ ] Accident and Emergency / Urgent care
- [ ] Other ____

Diagnosis – GP Entry

Not everyone will start their PF journey at the GP. By gathering information on the experiences of those who do, we can understand how this might impact the speed and ease at which individuals receive a confirmed PF diagnosis.

10 Before being referred for further tests, how many times did you/your loved one see a healthcare professional about the symptoms you/they had?

- ☐ None - went straight to hospital
- ☐ Saw a healthcare professional once
- ☐ Saw a healthcare professional twice
- ☐ Saw a healthcare professional 3 or 4 times
- ☐ Saw a healthcare professional 5 times or more
- ☐ Don't know/can't remember

11 After going to the GP with symptoms, how long was it before they agreed to refer you/your loved one to a respiratory consultant in hospital?

- ☐ Less than a month
- ☐ More than 1 month but less than 3 months
- ☐ More than 3 months but less than 6 months
- ☐ More than 6 months but less than a year
- ☐ More than a year
- ☐ Don't know/can't remember
- ☐ Saw a healthcare professional 3 or 4 times
- ☐ Saw a healthcare professional 5 times or more
- ☐ Don't know/can't remember

Diagnosis, Misdiagnosis and Being Heard

This section is intended to improve understanding of the delays and challenges faced in receiving a confirmed PF diagnosis. This will help to identify how this process can be more efficient for the benefit of patients, healthcare professionals, and the healthcare system as a whole.

12 Were you/your loved one diagnosed with another disease first which later turned out to be PF?

☐ Yes ☐ No ☐ Don't know/can't remember

13 If yes, which disease was PF initially misdiagnosed as?

____

14 If yes (to the question above), did you/they start treatment for another disease first?

☐ Yes ☐ No ☐ Don't know/can't remember ☐ Not applicable – no misdiagnosis

Action for Pulmonary Fibrosis

15 To what extent do you agree with the following statements?
If not applicable, please select 'don't know/can't remember'.

	Strongly Agree	Agree	Neutral	Disagree	Strongly Disagree	Don't know/can't remember
My/my loved one's initial health concerns were believed and taken seriously.	☐	☐	☐	☐	☐	☐
My/my loved one's initial health concerns were investigated in a timely manner.	☐	☐	☐	☐	☐	☐
I/my loved one did not face unnecessary delays in reaching a diagnosis.	☐	☐	☐	☐	☐	☐
The delays that were faced (in reaching diagnosis)could not have been avoided.	☐	☐	☐	☐	☐	☐

16 Approximately how long after your/your loved one's **first** visit to a healthcare professional about initial PF symptoms, did you/they receive an accurate diagnosis?

- ☐ Less than 1 month
- ☐ More than 1 month but less than 3 months
- ☐ More than 3 months but less than 6 months
- ☐ More than 6 months but less than a year
- ☐ More than a year but less than 2 years
- ☐ More than 2 years
- ☐ Don't know/can't remember

17 Once referred, how long did it take for you/your loved one to be seen by a respiratory consultant in a hospital?

- ☐ Less than 3 months
- ☐ More than 3 months but less than 6 months
- ☐ 7-12 months
- ☐ 1-2 years
- ☐ Over 2 years
- ☐ Not seen yet (still waiting)

Diagnosis, Misdiagnosis and Being Heard (continued)

18 How far do you/does your loved one live from the treating hospital?

- ☐ Less than 20 miles
- ☐ More than 20 miles but less than 50 miles
- ☐ More than 50 miles but less than 100 miles
- ☐ More than 100 miles
- ☐ Don't know/can't remember

19 How easy or difficult has it been for you/your loved one to travel to the treating hospital?

- ☐ Very easy
- ☐ Quite easy
- ☐ Neither easy nor difficult
- ☐ Quite difficult
- ☐ Very difficult
- ☐ Not applicable/ don't know/ can't remember

20 To what extent do you agree with the following statement?
"At the point of diagnosis, the information I/my loved one was provided with was...

	Strongly Agree	Agree	Neither Agree nor Disagree	Disagree	Strongly Disagree	Not sure/ can't remember
The right amount to receive at the time.	☐	☐	☐	☐	☐	☐
Useful for informing us about the condition.	☐	☐	☐	☐	☐	☐
Useful in telling us what to expect next.	☐	☐	☐	☐	☐	☐
Timely – the right information at the right time.	☐	☐	☐	☐	☐	☐

Treatment – Antifibrotic Drug Therapy

This is relevant to all PF patients and carers/loved ones and will ask you about receiving information and advice about antifibrotic drugs. This section will help us to understand the growing demand for antifibrotics, and how services might need to change to sustainably meet this demand.

21 Are you/is your loved one currently being prescribed antifibrotic medication?

☐ Yes ☐ No ☐ Not sure/can't remember

22 How soon after diagnosis did the healthcare provider inform you/your loved one about the potential for antifibrotic drug treatment? (Please note that antifibrotic drugs are not right for everyone, so not everyone will have had this mentioned by their clinician).

- ☐ Immediately – at diagnosis
- ☐ Less than 3 months
- ☐ More than 3 months but less than 6 months
- ☐ More than 6 months but less than a year
- ☐ More than a year
- ☐ Antifibrotics have never been discussed
- ☐ Not an appropriate treatment for my/their condition
- ☐ Don't know/can't remember

23 To what extent do you agree with the following statement about antifibrotics? "I/my loved one had enough information about..."

	Strongly Agree	Agree	Neither Agree nor Disagree	Disagree	Strongly Disagree	Not sure/ can't remember
The potential side-effects.	☐	☐	☐	☐	☐	☐
How to access the treatment.	☐	☐	☐	☐	☐	☐
The treatment plan and monitoring.	☐	☐	☐	☐	☐	☐
The effectiveness of the treatment.	☐	☐	☐	☐	☐	☐

24 Roughly how much time passed between being told antifibrotic treatment was needed and starting on this treatment?

- ☐ Less than 3 months
- ☐ More than 3 months but less than 6 months
- ☐ 7 - 12 months
- ☐ 1 - 2 years
- ☐ Over 2 years
- ☐ Still waiting
- ☐ Not applicable
- ☐ Don't know/can't remember

25 Which, if any, of the following side effects have you/your loved one experienced from antifibrotic treatment? Please leave blank if you have not had antifibrotics.

- ☐ Diarrhoea
- ☐ Nausea (feeling sick)
- ☐ Stomach pain
- ☐ Vomiting
- ☐ Liver problems
- ☐ Decreased appetite
- ☐ Headache
- ☐ Weight loss
- ☐ High blood pressure
- ☐ Other ____________

Treatment – Antifibrotic Drug Therapy (continued)

26 If you/your loved one had side effects from antifibrotics, which had the greatest impact on your/their life? Please leave blank if you have not had antifibrotics.

- ☐ Diarrhoea
- ☐ Nausea (feeling sick)
- ☐ Stomach pain
- ☐ Vomiting
- ☐ Liver problems
- ☐ Decreased appetite
- ☐ Headache
- ☐ Weight loss
- ☐ High blood pressure
- ☐ Other ___________

Symptom Management and Supportive Services

This section will ask you about being informed of supportive services and being able to access them. The first two questions are marked as mandatory as this is key to improving understanding of the PF care you/your loved one received. Your response to this section will help us to determine what support is being or not being provided across the UK.

27 Which supportive services did you or your loved one access? (If needed)

	No - I/they didn't need this	No - I/they couldn't access but it would have been helpful	Yes - referred by a healthcare professional	Yes - I/they had to find this personally	Don't know/can't remember
Information about pulmonary fibrosis	☐	☐	☐	☐	☐
A local or national support group	☐	☐	☐	☐	☐
Pulmonary rehabilitation	☐	☐	☐	☐	☐
Physiotherapy or Occupational Therapy	☐	☐	☐	☐	☐
Psychological support	☐	☐	☐	☐	☐
Oxygen services	☐	☐	☐	☐	☐
Dietitian	☐	☐	☐	☐	☐
End of life or advanced care planning	☐	☐	☐	☐	☐
Action for Pulmonary Fibrosis Support Line	☐	☐	☐	☐	☐

28 Were you/they able to access these services when you/they needed them?

	No - wasn't told about it	No - waiting times were too long	Yes - but had to wait longer than desired	Yes - in a timely fashion	Not applicable
Information about pulmonary fibrosis	☐	☐	☐	☐	☐
A local or national support group	☐	☐	☐	☐	☐
Pulmonary rehabilitation	☐	☐	☐	☐	☐
Physiotherapy or Occupational Therapy	☐	☐	☐	☐	☐
Psychological support	☐	☐	☐	☐	☐
Oxygen services	☐	☐	☐	☐	☐
Dietitian	☐	☐	☐	☐	☐
End of life or advanced care planning	☐	☐	☐	☐	☐
Action for Pulmonary Fibrosis Support Line	☐	☐	☐	☐	☐

29 If you experienced difficulties, what was the main type of barrier you/they faced when trying to access supportive services.

- ☐ Difficulty getting a referral
- ☐ Delays or issues after being referred (e.g. wait times, cancellations)
- ☐ Practical barriers (e.g. travel time, travel costs, appointment times)
- ☐ Physical barriers (e.g. mobility restrictions, accessibility issues)
- ☐ Not applicable - still waiting
- ☐ Not applicable - faced no access issues

30 Could you speak to a Specialist Nurse when needed?

- ☐ Yes - when needed
- ☐ Yes - but not always
- ☐ No
- ☐ Don't know/can't remember

31 To what extent has your/your loved one's diagnosis and treatment of pulmonary fibrosis negatively affected your overall mental health/emotional wellbeing?

- ☐ Severely
- ☐ To some extent
- ☐ Slightly
- ☐ Not at all
- ☐ Don't know/can't remember

32 Since your/your loved one's diagnosis, have you received mental health support from any of the following sources? (Select all that apply)

- ☐ A local or national support group
- ☐ Action for Pulmonary Fibrosis
- ☐ NHS mental health practitioner (e.g. counsellor/therapist)
- ☐ Private mental health practitioner (e.g. counsellor/therapist)
- ☐ None
- ☐ Not applicable

33 At any point during your care and treatment were you told about Action for Pulmonary Fibrosis – the national charity for pulmonary fibrosis in the UK?

☐ Yes ☐ No ☐ Don't know/can't remember

Branching Question – Accessing Specialist Components of Care (England only)

This question is mandatory as your answer will direct you to the set of questions that are most relevant to you. The next questions are specific to those receiving care in England only, as the set up for specialist ILD care provided in England is different compared to the other devolved nations. If you haven't received care in England, go to question 39.

34 Have you (or the person you care for) been referred to a specialist ILD (Interstitial Lung Disease) service by a healthcare professional? (Centres referred to as 'Specialist ILD centres' are only in England). Please also note that a referral to a specialist centre is not necessary for all patients.

- ☐ Yes – **go to Accessing Specialist Care (England only), question 35**
- ☐ No – **go to Coordination and Communication of care, question 39**
- ☐ I do not receive care in England – **go to Coordination and Communication of care, question 39**
- ☐ Don't know/can't remember – **go to Coordination and Communication of care, question 39**

Accessing Specialist Care (England only)

This section is for survey participants who reside in England and experienced PF care there. The questions below are intended to indicate the state of the nation in terms of how and when people are moving into specialist care (when needed).

35 If referred, were you (or the person you care for) able to access the specialist ILD service? (In England, this service is within an ILD Specialist Centre).

- ☐ Yes, without issues
- ☐ Yes, with a considerable wait time
- ☐ No, we were not able to attend
- ☐ Don't know/can't remember/ not applicable

Action for Pulmonary Fibrosis

36 Once referred to a specialist ILD centre, how long did you/they wait for your/their first appointment?

- ☐ Less than 1 month
- ☐ More than 1 month but less than 3 months
- ☐ More than 3 months but less than 6 months
- ☐ More than 6 months but less than 1 year
- ☐ More than 1 year
- ☐ Still waiting
- ☐ Don't know/can't remember

37 How would you rate your experience accessing specialist ILD care?

- ☐ Very easy
- ☐ Somewhat easy
- ☐ Neutral
- ☐ Not so easy
- ☐ Very difficult
- ☐ Still waiting
- ☐ Not sure/can't remember

38 If you experienced difficulties, what was the main type of barrier you/they faced when trying to access ILD specialist services?

- ☐ Difficulty getting a referral
- ☐ Delays or issues after being referred (e.g. wait times, cancellations)
- ☐ Practical barriers (e.g. travel time, travel costs, appointment times)
- ☐ Physical barriers (e.g. mobility restrictions, accessibility issues)
- ☐ Not applicable – still waiting
- ☐ Not applicable – faced no access issues

Coordination and Communication of care

This section is intended to help us understand if and how different sites and departments involved in provision of PF care work together. Your answers will help to identify the links and networks that require support and/or strengthening.

39 After receiving a confirmed PF diagnosis, did your/your loved one's GP then become involved in providing or supporting PF care?

- ☐ Yes, the GP was actively involved from the beginning
- ☐ Yes, but only after some time
- ☐ No, the GP was not involved at all
- ☐ Not sure/can't remember

*Required

40 How would you describe the GP's role in supporting or providing care, if they were involved? (If the GP was not involved, please select 'not applicable'.)

- ☐ GP worked closely with the specialist team and care felt well-coordinated
- ☐ GP supported some aspects of care (e.g. prescriptions, test follow-ups)
- ☐ GP had minimal involvement, and there were no problems with care
- ☐ GP had minimal involvement, and there were gaps or delays in care as a result
- ☐ GP was involved, but the support was unclear or unhelpful
- ☐ Not sure / can't remember
- ☐ Not applicable

41 Are you/is your loved one receiving care from multiple departments (e.g., rheumatology, cardiology) at the same time? This is a branching question and is mandatory in order to take you to the follow-up questions that are most relevant to your experience.*

- ☐ Yes – go to Coordination and Communication of Care Across Specialisms, question 42
- ☐ No – go to Research section, question 45
- ☐ Don't know/can't remember – go to Research section, question 45

Coordination and Communication of Care Across Specialisms

This section is relevant to those who are receiving / have received care across multiple departments (not just respiratory care). These questions are intended to indicate the standard of communication and coordination of care between departments for those living with PF. Your answers will help us to inform and encourage improvements.

42 How would you rate the communication between departments involved in your/your loved one's care?

☐ Excellent ☐ Very good ☐ Good ☐ Fair ☐ Poor

43 What issues, if any, were experienced regarding communication/coordination between departments?

- ☐ Scheduling or duplication of tests
- ☐ Sharing of test results
- ☐ Scheduling of appointments
- ☐ Sharing of information
- ☐ Accountability
- ☐ No issues that had a negative impact
- ☐ My/their care involved great coordination and communication
- ☐ Other ______________________

Action for Pulmonary Fibrosis

44 Which issue, if any, had the greatest impact on your experience?

- ☐ Scheduling or duplication of tests
- ☐ Sharing of test results
- ☐ Scheduling of appointments
- ☐ Sharing of information
- ☐ Accountability
- ☐ No issues
- ☐ Other ______________________

Research

This section is relevant to all survey participants (those living with PF and carers) and refers to all forms of 'research'. These insights will help to inform our efforts to increase and encourage access to research across the UK.

45 Would you be interested in taking part in research? This includes clinical trials, questionnaires, registries and other forms of research.

- ☐ Yes
- ☐ No
- ☐ Maybe
- ☐ Don't know

46 Have you ever been invited to take part in research? This may have been called a trial or a study. This is also relevant to those who have been/are caring for an individual with PF, as there is research on this experience.

- ☐ Yes – a clinical trial
- ☐ Yes – a questionnaire
- ☐ Yes – other ___________________
- ☐ No
- ☐ Don't know/can't remember
- ☐ Other ______________________

47 Did you take part in this research?

- ☐ Yes
- ☐ No
- ☐ Don't know/can't remember
- ☐ Not applicable

About you

This section will ask you to provide some personal details about yourself/your loved one. Your response will support data about the proportion of PF patients that fall into different demographic categories.

This will help us to understand and highlight how the experience of seeking PF care might differ between specific groups, and specific locations. This is particularly helpful in working to address inequalities in standards of care and access to services.

48 What is your age range? If you are a carer or loved one, please indicate the age the individual you know was diagnosed with PF.

- ☐ 25 or under
- ☐ 26-35
- ☐ 36-45
- ☐ 46-55
- ☐ 56-65
- ☐ 66-75
- ☐ 76 and over
- ☐ I prefer not to say

49 Where do you live? If you are a carer or loved one, please indicate where care is/was received by the individual with PF.

- ☐ Channel Islands
- ☐ England - Greater London
- ☐ England - South East
- ☐ England - South West
- ☐ England - East
- ☐ England - East Midlands
- ☐ England - West Midlands
- ☐ England - Yorkshire and Humberside
- ☐ England - North West
- ☐ England - North East
- ☐ Wales - Mid Wales
- ☐ Wales - North
- ☐ Wales - South East
- ☐ Wales - South West
- ☐ Scotland - North East
- ☐ Scotland - South East
- ☐ Scotland - South West
- ☐ Scotland - Highlands and Islands
- ☐ Northern Ireland

50 Which sex were you assigned at birth?
If you are a carer or loved one, please indicate that of the individual diagnosed with PF.

- ☐ Male
- ☐ Female
- ☐ I prefer not to say

Action for Pulmonary Fibrosis

51 What is your/your loved one's ethnic background?
Please note that the list below is not exhaustive, and if your identity is not listed please provide it in the text box below.

- ☐ Arab
- ☐ Asian or Asian British – Bangladeshi
- ☐ Asian or Asian British – Chinese
- ☐ Asian or Asian British – Indian
- ☐ Asian or Asian British – Pakistani
- ☐ Black or Black British – African
- ☐ Black or Black British – Caribbean
- ☐ Mixed – White & Asian
- ☐ Mixed – White & Black African
- ☐ Mixed – White & Black Caribbean
- ☐ White – English, Welsh, Scottish, Northern Irish or British
- ☐ White – Irish
- ☐ White – Gypsy or Irish Traveller
- ☐ Prefer not to say
- ☐ I prefer to be identified as ___________

Thank you and keep in touch!

We would like to offer our sincere thanks for taking the time to complete this survey. It is hugely appreciated. Please share this survey with others in your life who are affected by PF and encourage them to take part in the call for urgent change.

If you would like to receive news and information from Action for Pulmonary Fibrosis please give us your details below.

You do not have to provide your details and can leave this blank if you prefer.

52 First name ____

53 Surname ____

54 Email address ____

55 I am happy to be contacted about:

- ☐ APF Email newsletter
- ☐ Results of the survey
- ☐ Shaping the charity's work
- ☐ Shaping PF research
- ☐ Fundraising

Table 1 | Proportion of responses by country and region respectively.

Country / Region	Number of responses (%)
**England**	844 (78.58)
East	59 (6.93)
East Midlands	89 (10.59)
London	38 (4.52)
North East	59 (6.93)
North West	100 (11.84)
South East	207 (24.54)
South West	164 (19.44)
West Midlands	61 (7.22)
Yorkshire	67 (7.99
**Northern Ireland**	29 (2.60)
**Scotland**	108 (9.68)
**Wales**	84 (7.53)
**Undisclosed**	18 (1.61)

Table 2 | Age at diagnosis by pulmonary fibrosis subtype. * p-value < 0.05 resulting from Chi-square test comparing IPF with non-IPF-ILD cases.

	≤ 35	36-45	46-55	56-65	66-75	>75	Undisclosed
**All ILD**	0.27	1.98	4.06	19.75	39.22	34.27	0.45
**IPF**	0.14	0.97	2.76	17.38	40.00	38.21	0.55
**Non-IPF-ILD** (p<0.001) *	0.52	3.91	6.51	24.22	37.76	26.82	0.26
**Covid-19 ILD**	0.00	0.00	17.65	23.53	35.29	23.53	0.00
**CTD-ILD**	0.00	6.35	12.70	25.40	41.27	14.29	2.04
**HP**	1.35	1.35	8.11	29.73	39.19	20.27	0.00
**Idiopathic interstitial pneumonia**	0.00	4.95	2.97	18.81	35.64	37.62	0.00
**Occupational ILD**	4.00	4.00	4.00	20.00	24.00	44.00	0.00
**Other ILD**	0.00	2.94	0.00	32.35	41.18	20.59	2.94
**RA-ILD**	0.00	0.00	5.45	16.36	49.09	29.09	0.00
**Sarcoidosis**	0.00	20.00	6.67	46.67	6.67	20.00	0.00

PF: pulmonary fibrosis, IPF: idiopathic pulmonary fibrosis, Non-IPF-PF: Non-idiopathic pulmonary fibrosis, ILD: Interstitial lung disease, CTD ILD: connective tissue disease associated interstitial lung disease, RA-ILD: rheumatoid arthritis associated interstitial lung disease.

Table 3 | Age at diagnosis by pulmonary fibrosis subtype, by sex * indicates statistical significance ($p<0.05$) in age at diagnosis by sex. M: males, F: females.

	Sex	≤ 35	36-45	46-55	56-65	66-75	>75	Undisclosed
**All ILD**	**M**	0.34	0.84	2.53	16.86	39.63	38.95	0.84
	**F**	0.19	3.31	5.85	23.20	38.60	28.46	0.39
**IPF**	**M**	0.00	0.23	1.38	16.82	39.40	41.47	0.69
	**F**	0.35	2.10	4.90	18.53	40.91	32.87	0.35
**Non-IPF-ILD ***	**M**	1.27	2.55	5.73	17.20	40.76	32.48	0.00
	**F**	0.00	4.85	7.05	29.07	35.68	22.91	0.44
**Covid-19 ILD**	**M**	0.00	0.00	14.29	0.00	42.86	42.86	0.00
	**F**	0.00	0.00	20.00	40.00	30.00	10.00	0.00
**CTD-ILD**	**M**	0.00	5.00	5.00	20.00	55.00	15.00	0.00
	**F**	0.00	6.98	16.28	27.91	34.88	13.95	0.00
**Hypersensitivity pneumonitis ***	**M**	4.17	0.00	12.50	8.33	58.33	16.67	0.00
	**F**	0.00	2.00	6.00	40.00	30.00	22.00	0.00
**Idiopathic interstitial pneumonia**	**M**	0.00	2.33	4.65	16.28	34.88	41.86	0.00
	**F**	0.00	6.90	1.72	20.69	36.21	34.48	0.00
**Occupational ILD**	**M**	5.00	0.00	0.00	20.00	30.00	45.00	0.00
	**F**	0.00	20.00	20.00	20.00	0.00	40.00	0.00
**Other ILD**	**M**	0.00	6.67	0.00	26.67	40.00	26.67	0.00
	**F**	0.00	0.00	0.00	36.84	42.11	15.79	5.26
**RA-ILD**	**M**	0.00	0.00	10.00	10.00	40.00	40.00	0.00
	**F**	0.00	0.00	2.86	20.00	54.29	22.86	0.00
**Sarcoidosis**	**M**	0.00	12.50	0.00	50.00	12.50	25.00	0.00
	**F**	0.00	28.57	14.29	42.86	0.00	14.29	0.00

PF: pulmonary fibrosis, IPF: idiopathic pulmonary fibrosis, Non-IPF-PF: Non-idiopathic pulmonary fibrosis, ILD: Interstitial lung disease, CTD ILD: connective tissue disease associated interstitial lung disease, RA-ILD: rheumatoid arthritis associated interstitial lung disease.

Table 4 | Number of symptoms prior to diagnosis by broad pulmonary fibrosis groups. M: males, F: females. No statistical difference in number of symptoms by sex by subtype was observed.

	**Sex**	**Number of symptoms prior to diagnosis (%)**			
		**0**	**1**	**2**	**3+**
**All ILD**		9.68	40.74	30.74	18.83
	M	9.88	42.54	30.24	17.34
	F	9.40	38.53	31.65	20.41
**IPF**		10.88	43.35	30.22	15.54
	M	12.04	44.80	29.41	13.73
	F	8.72	40.83	31.65	18.81
**Non-IPF-ILD**		7.76	36.57	31.58	24.10
	M	4.32	36.69	32.37	26.62
	F	10.09	36.24	31.65	22.02
**Covid-19 ILD**		12.50	31.25	31.25	25.00
	M	28.57	14.29	14.29	42.86
	F	0.00	44.44	44.44	11.11
**CTD ILD**		9.52	26.98	41.27	22.22
	M	10.53	36.84	31.58	21.05
	F	9.30	23.26	46.51	20.93
**Hypersensitivity pneumonitis**		4.35	37.68	28.99	28.99
	M	0.00	42.86	33.33	23.81
	F	6.25	35.42	27.08	31.25
**Idiopathic interstitial pneumonia**		8.33	45.83	27.18	18.75
	M	2.63	44.74	34.21	18.42
	F	12.28	45.61	22.81	19.30
**Occupational ILD**		4.35	34.78	26.09	34.78
	M	0.00	33.33	27.78	38.89
	F	25.00	25.00	25.00	25.00
**Other ILD**		6.25	50.00	31.25	12.50
	M	7.14	42.86	35.71	14.29
	F	5.56	55.56	27.78	11.11
**RA-ILD**		12.24	26.53	30.22	15.54
	M	0.00	20.00	46.67	33.33
	F	17.65	29.41	29.41	23.53
**Sarcoidosis**		0.00	23.08	30.77	46.15
	M	0.00	28.57	14.29	57.14
	F	0.00	20.00	60.00	20.00

PF: pulmonary fibrosis, IPF: idiopathic pulmonary fibrosis, Non-IPF-PF: Non-idiopathic pulmonary fibrosis, ILD: Interstitial lung disease, CTD ILD: connective tissue disease associated interstitial lung disease, RA-ILD: rheumatoid arthritis associated interstitial lung disease.

Figure 1 | Symptom burden by pulmonary fibrosis subtypes, * indicates statistical difference ($p<0.05$) in percentage between IPF and the subtype. PF: pulmonary fibrosis, IPF: idiopathic pulmonary fibrosis, Non-IPF-PF: Non idiopathic pulmonary fibrosis, ILD: interstitial lung disease, CTD ILD: connective tissue diseases associate interstitial lung disease, RA-ILD: rheumatoid arthritis interstitial lung disease.

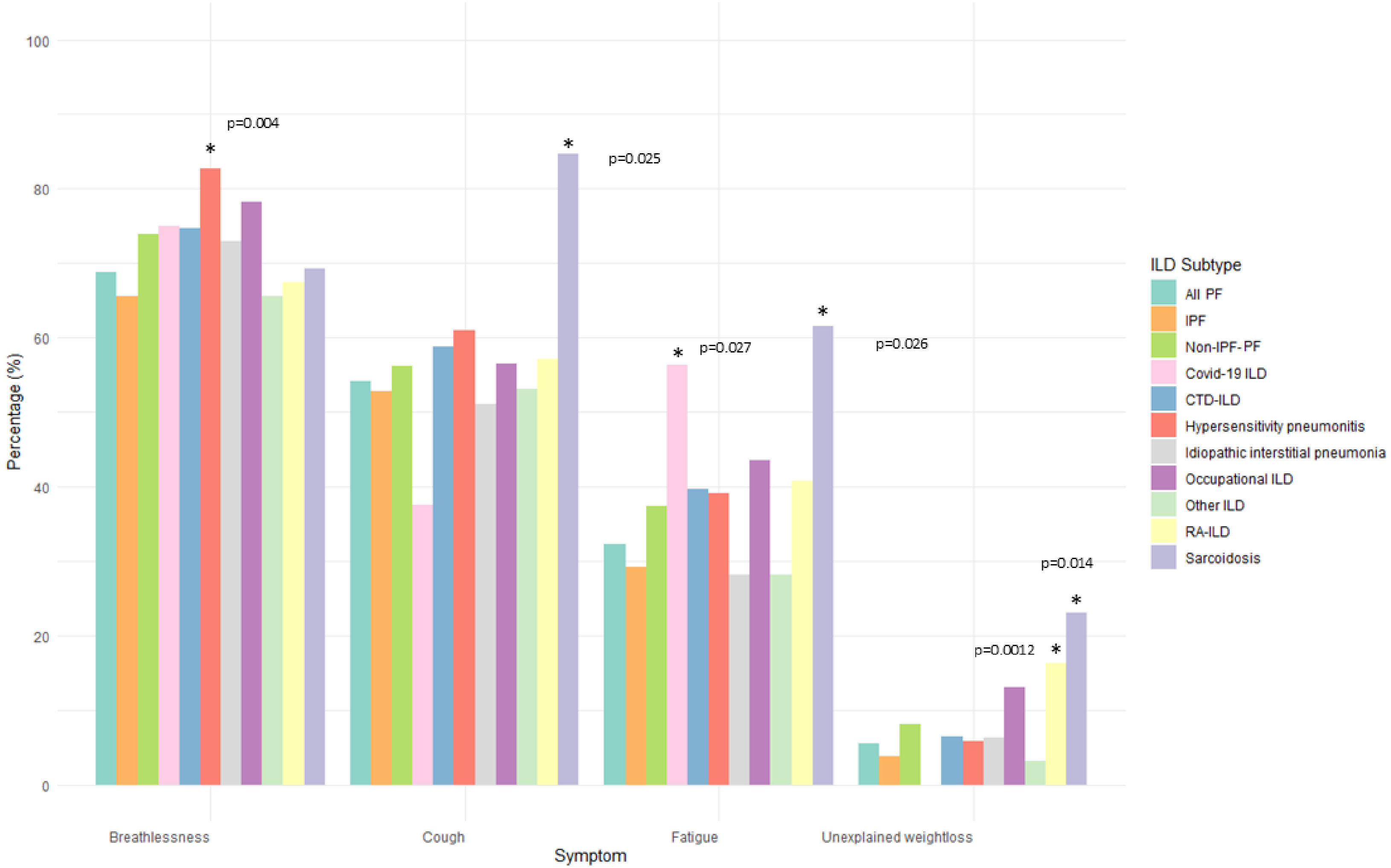

Table 5 | Percentage of people reporting symptoms prior to diagnosis of pulmonary fibrosis, stratified by ILD subtype and sex. * Indicates significant differences in percentages ($p<0.05$) between sex. M: males, F: females.

		Percentage of people with symptoms prior to diagnosis (%)			
	**Sex**	**Breathlessness**	**Cough**	**Fatigue**	**Unexplained weight loss**
**All ILD**	M	68.35	51.81	29.84	6.45
	F	69.50	56.88	35.09	4.13
**IPF**	M	63.87	50.42	26.89	4.76
	F	69.27	56.88	33.03	2.29
**Non-IPF-ILD**	M	79.86*	55.40	37.41	10.79
	F	69.72	56.88	37.16	5.96
**Covid-19 ILD**	M	57.14	57.14	57.14	0.00
	F	88.89	22.22	55.56	0.00
**CTD-ILD**	M	73.68	47.37	31.58	10.53
	F	74.42	62.79	41.86	2.33
**Hypersensitivity pneumonitis**	M	85.71	71.43	23.81	0.00
	F	81.25	56.25	45.83	8.33
**Idiopathic interstitial pneumonia**	M	76.32	50.00	31.58	13.16*
	F	70.18	52.63	26.32	1.75
**Occupational ILD**	M	83.33	61.11	44.44	16.67
	F	50.00	50.00	50.00	0.00
**Other ILD**	M	78.57	35.71	35.71	7.14
	F	55.56	66.67	22.22	0.00
**RA-ILD**	M	93.33*	53.33	53.33	13.33
	F	55.88	58.82	35.29	17.65
**Sarcoidosis**	M	85.71	85.71	57.14	28.57
	F	40.00	80.00	60.00	20.00

PF: pulmonary fibrosis, IPF: idiopathic pulmonary fibrosis, Non-IPF-PF: Non-idiopathic pulmonary fibrosis, ILD: Interstitial lung disease, CTD ILD: connective tissue disease associated interstitial lung disease, RA-ILD: rheumatoid arthritis associated interstitial lung disease.

Table 6 | Number of times people saw a healthcare professional regarding ILD symptoms prior to referral, including the median number of visits. * Indicates significant differences in percentages (p<0.05) between sex. † Indicates significant difference in the number of visits between the subgroup and IPF, p<0.05. M: males, F: females.

	**Sex**		**Saw a healthcare professional (%)**			
		**Median**	**Once**	**Twice**	**3-4 times**	**5+**
**All ILD**		2	34.59	21.54	26.73	17.14
	M*	2	39.88	24.28	23.70	12.14
	F	3	28.62	18.37	30.39	22.61
**IPF**		2	33.10	25.12	27.93	13.85
	M	2	37.11	25.00	27.34	10.55
	F	2	27.88	25.45	28.48	18.18
**Non-IPF-ILD**		2	37.62	14.29	24.29	23.81
	M *	2	47.78	22.22	13.33	16.67
	F	3	29.66	8.47	33.05	28.81
**Covid-19 ILD**		1	50.00	0.00	50.00	0.00
	M	1	100.00	0.00	0.00	0.00
	F	2	50.00	0.00	50.00	0.00
**CTD ILD †**		3	28.12	18.75	21.88	31.25
	M	2	40.00	30.00	10.00	20.00
	F	3	22.73	13.64	27.27	36.36
**Hypersensitivity pneumonitis**		3	31.82	13.64	29.55	25.00
	M*	1.5	50.00	25.00	0.00	25.00
	F	3	21.43	7.14	46.43	25.00
**Idiopathic interstitial pneumonia**		2	3.33	17.46	23.81	25.40
	M	2	45.16	22.58	12.90	19.35
	F	3	21.88	12.50	34.38	31.25
**Occupational ILD**		1.5	50.00	14.29	21.43	14.29
	M	1.5	50.00	16.67	16.67	16.67
	F	2	50.00	0.00	50.00	0.00
**Other ILD**		3	47.37	0.00	36.84	15.79
	M	3	33.33	0.00	50.00	16.67
	F	1	53.85	0.00	30.77	15.38
**RA-ILD**		1	51.72	17.24	13.79	17.24
	M	1	58.33	33.33	8.33	0.00
	F	2	47.06	5.88	17.65	29.41
**Sarcoidosis**		3.5	25.00	0.00	25.00	50.00
	M	2	20.00	0.00	50.00	0.00
	F	4	0.00	0.00	0.00	100.00

PF: pulmonary fibrosis, IPF: idiopathic pulmonary fibrosis, Non-IPF-PF: Non-idiopathic pulmonary fibrosis, ILD: Interstitial lung disease, CTD ILD: connective tissue disease associated interstitial lung disease, RA-ILD: rheumatoid arthritis associated interstitial lung disease.

Table 7 | Responses to the statement *"My initial health concerns were believed and taken seriously"* by sex. Using the data of people currently living with pulmonary fibrosis. * indicates statistically significant differences in responses between sexes, p<0.05.

Response	All responses (%)	Male (%)	Female (%)*
**Agree- I felt listened to**	68.44	74.40	61.94
**Neutral**	12.78	13.88	11.58
**Disagree – I did not feel listened to**	18.78	11.71	26.48

Table 8 | Responses to the statement *"The delays faced in reaching a diagnosis could not have been avoided"* by sex. Using the data of people currently living with pulmonary fibrosis.

Response	All responses (%)	Male (%)	Female (%)
**Agree**	40.23	42.82	37.47
**Neutral**	23.70	24.69	22.64
**Disagree**	36.07	32.49	39.89

Table 9 | Proportion of people who were misdiagnosed with another condition. † Of the people who were misdiagnosed, the percentage of people who received treatment for the misdiagnosed condition. * Indicates significant differences in percentages (p<0.05) between subtype and IPF. M: males, F: females.

	Sex	Percentage misdiagnosed	Received treatment for misdiagnosed condition (%) †
**All ILD**		32.96	70.82
	M	32.81	72.57
	F	33.13	68.83
**IPF**		30.90	67.00
	M	31.04	68.85
	F	30.69	64.10
**Non-IPF-ILD**		36.83	76.84
	M	37.75	81.13
	F	36.20	73.68
**Covid-19 ILD**		14.29	100.00
	M	0.00	0.00
	F	20.00	100.00
**CTD ILD**		36.07	77.27
	M	35.00	85.71
	F	36.59	73.33
**Hypersensitivity pneumonitis ***		43.06	76.67
	M	34.78	85.71
	F	46.94	73.91
**Idiopathic interstitial pneumonia**		35.71	75.00
	M	38.10	75.00
	F	33.93	75.00
**Occupational ILD ***		56.00	64.29
	M	55.00	63.64
	F	60.00	66.67
**Other ILD**		44.12	85.71
	M	33.33	100.00
	F	52.63	80.00
**RA-ILD**		26.42	83.33
	M	36.84	100.00
	F	20.59	66.67
**Sarcoidosis**		26.67	66.67
	M	37.50	100.00
	F	14.29	0.00

Table 10 | Distribution of misdiagnosed conditions by sex.

Misdiagnosed condition	Number of people	Male (%)	Female (%)
**Asthma**	62	43.55	56.45
**COPD**	47	53.19	46.81
**Cardiovascular problems**	35	37.14	62.86
**Pneumonia**	25	44.00	56.00
**Infection**	46	54.35	45.65

Table 11 | Side effects experienced by people receiving antifibrotics by sex.

* Indicates significant differences in percentages (p<0.05) with respect to sex.

	Total (%)	Males (%)	Females (%)
**Asymptomatic**	9.35	8.53	10.88
**One side effect**	15.51	16.47	13.99
**Multiple side effects**	75.14	75.00	75.13
**Diarrhoea**	58.40	60.00	54.92
**Decreased appetite**	52.05	52.65	50.78
**Nausea ***	50.00	42.06	63.73
**Weight loss**	41.04	35.23	44.12
**Stomach pain**	32.46	29.71	36.79
**Vomiting ***	17.72	11.76	27.98
**Headaches ***	14.18	10.29	20.73
**Liver problems**	5.97	4.71	8.29
**High blood pressure**	4.85	3.82	6.74
**Sun sensitivity**	1.31	1.47	1.04

Table 12 | Side effects experienced by people receiving antifibrotics by age at diagnosis. * Indicates significant differences in percentages (p<0.05) with respect to age.

	Age at diagnosis (%)		
	**≤ 65 years**	**66-75 years**	**>75 years**
**Asymptomatic**	8.70	8.29	10.05
**One side effect**	12.17	16.13	17.59
**Multiple side effects**	79.13	75.58	72.36
**Diarrhoea ***	68.70	54.84	56.78
**Decreased appetite**	58.26	48.85	52.76
**Nausea**	56.52	51.61	45.23
**Weight loss**	41.74	37.33	44.72
**Stomach pain**	35.65	35.48	28.14
**Vomiting**	20.87	19.35	14.57
**Headaches**	19.13	15.67	10.05
**Liver problems**	10.43	4.61	5.03
**High blood pressure**	5.22	5.07	4.52
**Sun sensitivity**	1.74	0.92	1.51

Appendix 1d

STROBE Statement—checklist of items that should be included in reports of observational studies

	Item No.	Recommendation	Page No.	Relevant text from manuscript
**Title and abstract**	1	(*a*) Indicate the study's design with a commonly used term in the title or the abstract	2	*Design*<br>A cross-sectional analysis of the 2025 PF State of the Nation Survey, conducted by Action for Pulmonary Fibrosis.
		(*b*) Provide in the abstract an informative and balanced summary of what was done and what was found	2	Using the survey results which included information from 1,270 individuals (73.78% patients) with lived experience of PF. Differences in symptoms, diagnostic pathways and patient experience were examined using Chi-squared and Fishers exact tests.<br><br>Respondents with idiopathic pulmonary fibrosis (IPF) were more likely to be male, whilst female dominance was observed in those reporting diagnosis of non-IPF-PF. Males were diagnosed at an older age than females, were referred to respiratory services more quickly, and had fewer primary care appointments prior to referral. More than 90% of the cohort reported symptoms prior to diagnosis, but symptomology differed by PF subtype; breathlessness was more commonly reported by those with hypersensitivity pneumonitis (p=0.004) and cough was more commonly reported by people with sarcoidosis (p=0.025). Almost one quarter (24.93%) of people reported symptoms to a physician only after experiencing them for at least one year. Treatment burden was high, antifibrotic side effects were common and differences with respect to age and sex were observed.
**Introduction**				
Background/rationale	2	Explain the scientific background and rationale for the investigation being reported	3	Despite improvements in epidemiological evidence of PF, gaps in the knowledge base remain with respect to disease symptomology and how this differs between subtypes as well as sex. It is known that side effects of current

				antifibrotic medication can lead to early discontinuation and may negatively impact quality of life due to gastrointestinal problems and fatigue [9–11]. It is not currently well understood if side effects of antifibrotics differ by age or sex. Systematic, representative and well-designed research is pivotal to guide understanding of symptomology and treatment plans leading to improved care for people with PF.
Objectives	3	State specific objectives, including any prespecified hypotheses	3	aim of gathering the perspectives of patients, carers and bereaved individuals with respect to perceived experiences of PF care
**Methods**				
Study design	4	Present key elements of study design early in the paper	4	Supplementary appendix 1A and 1B
Setting	5	Describe the setting, locations, and relevant dates, including periods of recruitment, exposure, follow-up, and data collection	4	The Pulmonary Fibrosis State of the Nation lived experience Survey was deployed in four languages (English, Welsh, Polish and Punjabi) on the 21/05/2025 and closed submissions on the 30/06/2025[12].
Participants	6	(*a*) *Cohort study*—Give the eligibility criteria, and the sources and methods of selection of participants. Describe methods of follow-up<br><br>*Case-control study*—Give the eligibility criteria, and the sources and methods of case ascertainment and control selection. Give the rationale for the choice of cases and controls<br><br>*Cross-sectional study*—Give the eligibility criteria, and the sources and methods of selection of participants	NA	NA
		(*b*) *Cohort study*—For matched studies, give matching criteria and number of exposed and unexposed<br><br>*Case-control study*—For matched studies, give matching criteria and the number of controls per case	NA	NA
Variables	7	Clearly define all outcomes, exposures, predictors, potential confounders, and effect modifiers. Give diagnostic criteria, if applicable	4	Where possible we describe the responses across self-reported subtypes of PF. These are: idiopathic pulmonary fibrosis (IPF), rheumatoid arthritis associated interstitial lung disease (RA-ILD), connective tissue disease associated interstitial lung disease (CTD-ILD), hypersensitivity pneumonitis (HP), COVID-19 interstitial lung disease, idiopathic interstitial pneumonia (IIP), occupational PF (which included silicosis and asbestosis), pulmonary sarcoidosis and Other PF (including combined pulmonary fibrosis and emphysema (CPFE), drug induced PF (D-ILD), familial

				pulmonary fibrosis, fibrosing organising pneumonia, radiation induced pulmonary fibrosis and progressive pulmonary fibrosis). We also compared the perspectives of people with IPF with those of people with non-IPF-PF.
Data sources/ measurement	8*	For each variable of interest, give sources of data and details of methods of assessment (measurement). Describe comparability of assessment methods if there is more than one group	*5*	We described the distribution of respondents across pulmonary fibrosis (PF) subtypes and examined differences by sex (male/ female) and age at diagnosis (25 or under, 26-25, 36-45, 46-55, 56-65, 66-75, 76+).
Bias	9	Describe any efforts to address potential sources of bias	6	Throughout this report some analyses were conducted using the data of people with current PF (patients) to minimise bias, whilst no restrictions were applied for other questions. Where necessary the specific group of people whose data were analysed is clearly stated.
Study size	10	Explain how the study size was arrived at	6	Throughout this report some analyses were conducted using the data of people with current PF (patients) to minimise bias, whilst no restrictions were applied for other questions. Where necessary the specific group of people whose data were analysed is clearly stated.

Continued on next page

Quantitative variables	11	Explain how quantitative variables were handled in the analyses. If applicable, describe which groupings were chosen and why	N/A all were categorical
Statistical methods	12	(*a*) Describe all statistical methods, including those used to control for confounding	Confounding was not analysed as this was descriptive, all other methods are described
		(*b*) Describe any methods used to examine subgroups and interactions	Mentioned throughout methods and discussion
		(*c*) Explain how missing data were addressed	Numbers of missing data were reported for each analysis, these datapoints were not included in the respective analyses.
		(*d*) *Cohort study*—If applicable, explain how loss to follow-up was addressed<br>*Case-control study*—If applicable, explain how matching of cases and controls was addressed<br>*Cross-sectional study*—If applicable, describe analytical methods taking account of sampling strategy	N/A NA
		(*e*) Describe any sensitivity analyses	N/A NA
**Results**			
Participants	13*	(a) Report numbers of individuals at each stage of study—eg numbers potentially eligible, examined for eligibility, confirmed eligible, included in the study, completing follow-up, and analysed	Supplementary material tables
		(b) Give reasons for non-participation at each stage	Supplementary material tables
		(c) Consider use of a flow diagram	This was considered and deemed not possible in this instance
Descriptive data	14*	(a) Give characteristics of study participants (eg demographic, clinical, social) and information on exposures and potential confounders	Presented in tables and described in opening paragraph of results
		(b) Indicate number of participants with missing data for each variable of interest	Present in supplementary materials
		(c) *Cohort study*—Summarise follow-up time (eg, average and total amount)	N/A
Outcome data	15*	*Cohort study*—Report numbers of outcome events or summary measures over time	*N/A*
		Case-control study—Report numbers in each exposure category, or summary measures of exposure	*N/A*
		Cross-sectional study—Report numbers of outcome events or summary measures	*Provided in tables*
Main results	16	(*a*) Give unadjusted estimates and, if applicable, confounder-adjusted estimates and their precision (eg, 95% confidence interval). Make clear which confounders were adjusted for and why they were included	N/A
		(*b*) Report category boundaries when continuous variables were categorized	N/A
		(*c*) If relevant, consider translating estimates of relative risk into absolute risk for a meaningful time period	N/A

Continued on next page

Other analyses	17	Report other analyses done—eg analyses of subgroups and interactions, and sensitivity analyses	All analyses are presented in either the main body or supplement
**Discussion**			
Key results	18	Summarise key results with reference to study objectives	First paragraph of the discussion
Limitations	19	Discuss limitations of the study, taking into account sources of potential bias or imprecision. Discuss both direction and magnitude of any potential bias	Discussed extensively in the limitations section of the art
Interpretation	20	Give a cautious overall interpretation of results considering objectives, limitations, multiplicity of analyses, results from similar studies, and other relevant evidence	Throughout the discussion
Generalisability	21	Discuss the generalisability (external validity) of the study results	The study achieved the largest response to date for a UK-based survey of lived experience with PF, providing a broad UK sample and supporting the generalisability of the findings within the UK PF population. Inclusion of both patients and carers also provided a broader perspective on the diagnostic journey.
**Other information**			
Funding	22	Give the source of funding and the role of the funders for the present study and, if applicable, for the original study on which the present article is based	N/A

*Give information separately for cases and controls in case-control studies and, if applicable, for exposed and unexposed groups in cohort and cross-sectional studies.

Note: An Explanation and Elaboration article discusses each checklist item and gives methodological background and published examples of transparent reporting. The STROBE checklist is best used in conjunction with this article (freely available on the Web sites of PLoS Medicine at http://www.plosmedicine.org/, Annals of Internal Medicine at http://www.annals.org/, and Epidemiology at http://www.epidem.com/). Information on the STROBE Initiative is available at www.strobe-statement.org.
